# Advances in CAR-T therapy for central nervous system tumors

**DOI:** 10.1186/s40364-024-00679-6

**Published:** 2024-11-06

**Authors:** Delian Zhou, Xiaojian Zhu, Yi Xiao

**Affiliations:** Department of Hematology, Tongji Hospital, Tongji Medical College, Huazhong University of Science and Technology, Wuhan, Hubei 430030 China

**Keywords:** Central nervous system tumors, Chimeric antigen receptor T-cell therapy, Glioblastoma, Medulloblastoma, Central nervous system lymphoma, Central nervous system acute lymphoblastic leukemia, Brain tumors

## Abstract

The application of chimeric antigen receptor T-cell therapy in central nervous system tumors has significantly advanced; however, challenges pertaining to the blood-brain barrier, immunosuppressive microenvironment, and antigenic heterogeneity continue to be encountered, unlike its success in hematological malignancies such as acute lymphoblastic leukemia and diffuse large B-cell lymphomas. This review examined the research progress of chimeric antigen receptor T-cell therapy in gliomas, medulloblastomas, and lymphohematopoietic tumors of the central nervous system, focusing on chimeric antigen receptor T-cells targeting antigens such as EGFRvIII, HER2, B7H3, GD2, and CD19 in preclinical and clinical studies. It synthesized current research findings to offer valuable insights for future chimeric antigen receptor T-cell therapeutic strategies for central nervous system tumors and advance the development and application of this therapeutic modality in this domain.

## Introduction

 The principle of chimeric antigen receptor T-cell (CAR-T) therapy involves genetically modifying T cells to specifically recognize distinct targets on the tumor cell surface, thereby inducing cytotoxic effects. CAR-T therapy has become a major focus of immunotherapy research for malignant tumors in recent years, representing a milestone breakthrough in targeted therapy [1]. CAR-T therapy has shown success in treating various refractory or recurrent hematological malignancies and is considered a potentially curative approach for certain oncological conditions [2, 3]. However, its application in solid tumors, particularly central nervous system (CNS) tumors, remains limited owing to the complex tumor microenvironment (TME) of CNS tumors and the presence of the blood-brain barrier (BBB), introducing significant challenges for CAR-T therapy applications. Researchers are actively investigating various strategies to overcome these challenges, and various clinical and preclinical studies have established a foundation for the future development of CAR-T therapy for CNS tumors.

## Challenges of CAR-T therapy in CNS tumors

### Blood-brain barrier

**Fig. 1:**
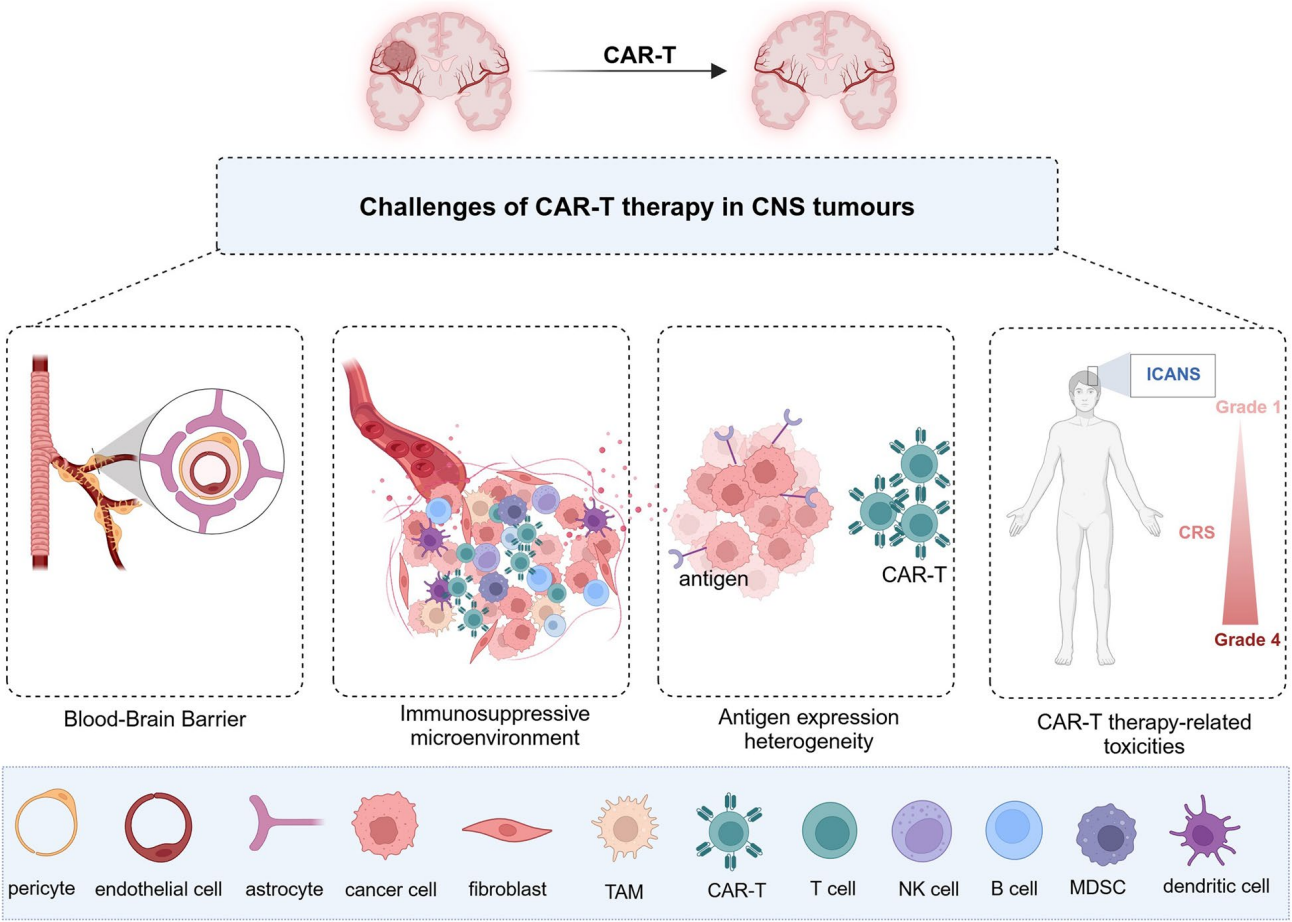
Challenges of CAR-T therapy in CNS tumors. The blood-brain barrier, immunosuppressive microenvironment, antigen expression heterogeneity, and CAR-T therapy-related toxicity are major challenges in CAR-T cell therapy for central nervous system tumors

The BBB is a highly selective, semi-permeable barrier separating the cerebral blood vessels and brain tissue (Fig. 1). The BBB comprises endothelial cells, a basement membrane, and astrocytic end feet. The main functions of the BBB include protecting brain tissue, maintaining CNS homeostasis, and regulating the passage of essential nutrients and oxygen while preventing the entry of harmful substances [4]. The tight junctions and adherens junctions between brain capillary endothelial cells form the physical barrier, limiting paracellular transport into the brain. Thus, molecules in the bloodstream must traverse both the apical and basolateral membranes of endothelial cells to access the brain. Additionally, efflux proteins, such as P-glycoprotein, multidrug resistance protein (MRP), and breast cancer resistance protein (BCRP), on endothelial cells form a biochemical barrier by actively transporting exogenous substances back into the systemic circulation, limiting their accumulation in the brain [5]. However, this selectivity impedes the delivery of drugs and therapeutic agents to the brain [6, 7]. Inflammation, infection, and cancer can compromise BBB integrity, leading to immune cell infiltration and accumulation within the CNS [8]. For instance, in gliomas, reduced expression of tight junction proteins and overexpression of aquaporin-4 impair the integrity of endothelial cell junctions [9]. Currently, the primary methods for delivering CAR-T cells to the brain include intravenous injection, local intracerebroventricular delivery, and intracavitary tumor injection [10]. Intravenous injection is the most common route in treatment and clinical trials, yet it poses significant challenges for CNS tumors, as CAR-T cells often struggle to infiltrate the TME. Intracerebroventricular administration involves direct injection of CAR-T cells into the ventricular system, facilitating their distribution into the cerebrospinal fluid. Intracerebroventricular injection was first employed in CAR-T cell therapy for recurrent GBM. Intracavitary tumor injection involves direct CAR-T cell administration into the tumor mass [11]. In contrast to intravenous injections, local delivery methods can transport CAR-T cells directly to the tumor site, bypassing the BBB. This approach enhances CAR-T cell tumor infiltration and anti-tumor activity, thereby improving therapeutic outcomes, especially in glioblastoma and brain metastases from breast cancer [12–14].

### Immunosuppressive microenvironment

The tumor immunosuppressive microenvironment weakens the ability of immune cells to attack tumors via diverse mechanisms. This microenvironment comprises various immunosuppressive components, such as tumor-associated macrophages (TAMs)/microglia, regulatory T cells (Tregs), and myeloid-derived suppressor cells (MDSCs) (Fig. 1). These cells diminish the anti-tumor activity of effector T cells by releasing inhibitory factors or directly suppressing their function. In addition, the extracellular matrix (ECM) physically obstructs T-cell infiltration and promotes immunosuppression via signaling pathways, further facilitating tumor immune escape [15–17].

TAMs/microglia are crucial components of the CNS TME and can be classified into two subtypes: M1 and M2. M1 represents a pro-inflammatory phenotype with anti-tumor effects, whereas macrophages recruited to the TME are usually polarised to the M2 type, producing anti-inflammatory cytokines promoting immunosuppression, angiogenesis, and tumor progression [18]. Additionally, microglia within glioblastoma (GBM) microenvironments frequently experience high oxidative stress, triggering an imbalance in lipid metabolic homeostasis via the NR4A2/SQLE pathway and subsequently impairing antigen-presenting capacity [19]. This process diminishes cytotoxic T lymphocyte (CTL) cell infiltration and impairs their cytotoxic function, further exacerbating the immunosuppressive microenvironment and promoting GBM tumor growth. MDSCs are limited in number within the CNS; nevertheless, they actively contribute to the initiation and regulation of immunosuppressive functions [20], primarily through mechanisms that include promoting tumor angiogenesis, inhibiting M1 macrophage polarisation, suppressing dendritic cell (DC) antigen presentation, and reducing natural killer (NK) cytotoxicity and T-cell activation [21]. MDSCs exert their immunosuppressive effects through multiple mechanisms, including the secretion of exosomes, the activity of pro-inflammatory cytokines (such as IL-13, IL-4, PGE2, IFN-γ, and IL-1β), and Toll-like receptor signaling pathways [21]. Additionally, immunosuppressive factors, including nitric oxide (NO), reactive oxygen species (ROS), and peroxynitrite (PNT), play significant roles in the immunosuppressive mechanisms employed by MDSCs [22]. Tumors promote the recruitment of MDSCs by secreting specific chemokines. For instance, chemokine axes such as C-X-C chemokine receptor type 4-C-X-C motif chemokine ligand 12 (CXCR4-CXCL12), CXCR2-CXCL5/8, and C-C chemokine receptor type 2- C-C motif chemokine ligand 2 (CCR2-CCL2) play a crucial role in this process [23]. Furthermore, IL-8 is regarded as a significant inducer of MDSC mobilization [24]. Studies have demonstrated that an increased number of MDSCs in GBM correlates with poorer patient prognosis [25]. Tregs can suppress the immune response in the tumor microenvironment and promote tumor development [26]. The primary mechanisms through which Tregs mediate immune suppression in the tumor microenvironment include the following: First, Tregs upregulate immune checkpoint receptors, including cytotoxic T-lymphocyte-associated protein 4 (CTLA-4), thereby inhibiting the interaction between CD80 and CD86 on antigen-presenting cells and the co-stimulatory receptor CD28 on effector T cells. Second, Tregs secrete a variety of immunosuppressive cytokines, including IL-10, IL-35, and transforming growth factor-beta (TGF-β), and express the high-affinity α subunit of the IL-2 receptor (CD25), effectively depleting IL-2 and subsequently suppressing the activation and survival of effector T cells. Furthermore, Tregs exhibit elevated expression of ecto-nucleotidases CD39 and CD73 on their surface, which further enhances immune suppression in the tumor microenvironment [27], thereby undermining anti-tumor immune responses.

Pervasive TGF-β expression in the immunosuppressive microenvironment can promote tumor cell proliferation and enhance their invasiveness [28]. Additionally, TGF-β can modulate the composition and function of immune cells, thereby facilitating tumor cell evasion of the host immune system [29, 30]. Myeloid cells promote tumor immune escape by releasing interleukin-10 (IL-10), which accumulates within mesenchymal-like tumor regions and results in T-cell depletion. Studies have shown that JAK/STAT pathway inhibition restores T-cell function, highlighting the critical role of IL-10 in the immunosuppressive microenvironment of GBM [31]. Chemokines directly target non-immune cells in the tumor microenvironment (e.g., tumor and vascular endothelial cells) and play critical roles in regulating tumor cell proliferation, preserving cancer stem cell characteristics, and promoting tumor invasion and metastasis [32]. A significant elevation in cerebrospinal fluid and serum levels of CCL2 was observed in patients with CNS tumors undergoing HER2 CAR-T cell therapy. This cytokine is known for its role in recruiting Tregs and MDSCs and contributes to the attenuation of CAR-T cell-mediated tumor cell destruction [33]. Interactions between tumor cells and the microenvironment play a crucial role in tumor cell proliferation, migration, and drug resistance in GBM. Hypoxic regions of GBM attract and sequester TAMs and CTL, resulting in immunosuppression [34]. Additionally, single-cell transcriptomic analysis revealed that specific tumor subpopulations promote brain-wide proliferation through synaptic connections with neurons. This mechanism suggests that neuronal activity induces tumor microtubule formation, thereby accelerating tumor invasion and dissemination [35]. These mechanisms synergistically enable tumor cells to evade immune surveillance, fostering their growth and dissemination.

### Antigen expression heterogeneity

Tumor targets can be classified into tumor-specific antigens (TSAs) and tumor-associated antigens (TAAs). TSAs are antigens that are exclusively expressed on tumor cells and not on normal tissues. Although these antigens are deemed ideal targets for CAR-T therapy, their expression on the surface of tumor cells is exceedingly rare, leading to significant limitations in clinical application. In contrast, TAAs are antigens that exhibit significantly higher expression levels in tumor cells than in normal tissues [36]. Currently, the vast majority of CAR-T products target TAAs, such as CD19, CD20, CD22, CD30, and BCMA in hematologic malignancies [37–39], as well as CEA, EGFR, HER2, EPHA2, and IL-13Rα2 in solid tumors [40]. Specific antigens play important roles in CAR-T cell functioning, however, antigen expression heterogeneity results in a limited number of antigens that can be targeted by CAR-T cells [41]. The expression levels of targets on tumor cell surfaces may fluctuate between patients and different time points or regions in the same patient, and primary and recurrent tumors show significantly different characteristics, demonstrating significant heterogeneity (Fig. 1) [42]. For instance, in a study investigating the efficacy of the epidermal growth factor receptor variant III (EGFRvIII) peptide vaccine Rindopepimut in treating GBM, it was observed that 21 patients (57%) experienced a loss of EGFRvIII expression after treatment; similarly, 23 out of 39 patients (59%) in the control group also exhibited the same outcome [43]. These results underscore the significant impact of antigen heterogeneity on targeted therapies. Furthermore, there exists a similar issue regarding declining antigen expression in CAR-T cell therapy. A study indicated that among patients receiving EGFRvIII CAR-T therapy, 71.4% exhibited reduced levels of antigen expression, while the growth of EGFRvIII-negative tumor cells was also observed [44]. These findings further emphasize the complexities of antigen heterogeneity during the therapeutic process. Additionally, the presence of cancer stem cells contributes to the extensive heterogeneity observed in GBM [45]. Therefore, ideal antigenic candidates for CAR-T therapy should be highly and uniformly expressed in tumor cells, with low inter-tumor heterogeneity [46], and should have minimal or almost no expression in normal tissues to improve treatment specificity and efficacy.

### CAR-T therapy-related toxicities

Toxic reactions of CAR-T therapy for CNS tumors pose significant challenges, particularly cytokine release syndrome (CRS) and immune effector cell-associated neurotoxicity syndrome (ICANS) (Fig. 1) [47, 48]. Clinical manifestations of ICANS include delirium, somnolence, aphasia, and tremor. Severe cases can present with seizures, cerebral edema, coma, or even death, with a fatality rate of approximately 3% [49]. The symptoms of ICANS are often similar to those of primary brain tumors, potentially causing diagnostic challenges.

Studies have shown that ICANS onset is closely related to high levels of cytokines such as IL-1, IL-6, and granulocyte-macrophage colony-stimulating factor (GM-CSF) in the cerebrospinal fluid (CSF), which are primarily released by activated monocytes/macrophages [50]. CRS can be effectively controlled through monocyte depletion and administration of the IL-6 receptor blocker tocilizumab or the IL-1 receptor blocker anakinra; however, these measures have failed to completely prevent the development of fatal neurotoxicity [51]. Furthermore, endothelial cell activation triggered by CAR-T therapy results in increased vascular permeability, which is further exacerbated by BBB disruption by CNS tumors. Multiple cytokines, such as IL-6 and interferon-γ (IFN-γ), can selectively pass through the compromised BBB and exacerbate ICANS outcomes [52, 53]. Therefore, management and control of toxic reactions during CAR-T therapy are crucial for achieving therapeutic success.

In addition to the previously mentioned challenges, CAR-T cell therapy encounters several obstacles related to technical processes in its practical application. First, the complexity of CAR design poses a substantial challenge, particularly regarding the selection of appropriate antigen targets, optimization of co-stimulatory molecule combinations, and enhancement of CAR-T cell specificity and persistence. Second, the complexity of the production process further restricts the widespread application of CAR-T therapy, involving in vitro expansion of CAR-T cells, the risk of insertional oncogenesis from genetic modification [54], and the transfection efficiency of CAR-T cells [55]. Furthermore, during treatment, the selection of CAR-T cell dosage, evaluation of therapeutic efficacy, and monitoring of side effects necessitate further research and standardization. These challenges within the technical processes not only affect efficacy and application but also contribute to elevated treatment costs, presenting another significant barrier to the advancement of CAR-T cell therapy [56]. To address these challenges, ongoing innovation and improvement are essential to ensure the broader application of CAR-T therapy.

## CAR-T therapy for CNS tumors

A wide variety of CNS tumors exist, nevertheless, this article focused on advances in CAR-T therapy for the following tumor types: GBM, diffuse midline glioma (DMG), diffuse intrinsic pontine glioma (DIPG), and ventricular meningiocytoma among gliomas; medulloblastoma among embryonal tumors; and lymphohematopoietic tumors involving the CNS. Table 1 lists relevant clinical trials that enrolled patients in recent years, with data from https://clinicaltrials.gov/.

**Table 1:** Ongoing clinical trials recruiting patients for CAR-T therapy in Central Nervous System tumors (https://clinicaltrials.gov/)

NCT	Study Title	Phase	Disease type	Target	Participants	Method of delivery	Research Institutions	Study Start	Status
NCT06186401	Anti-EGFRvIII synNotch Receptor Induced Anti-EphA2/​IL-13Ralpha2 CAR (E-SYNC) T Cells	phase 1	GBM	EGFRvIII; IL13Rα2; EphA2	20	IV	University of California	2024	Recruiting
NCT05802693	A Study to Evaluate the Safety, Tolerance and Initial Efficacy of EGFRvIII CAR-T on Glioblastoma	Early phase 1	GBM	EGFRvIII	22	ICV	Beijing Tsinghua Chang Gung Hospital	2023	Not yet recruiting
NCT05063682	The Efficacy and Safety of Brain-targeting Immune Cells (EGFRvIII-CAR T Cells) in Treating Patients With Leptomeningeal Disease From Glioblastoma. Administering Patients EGFRvIII -CAR T Cells May Help to Recognize and Destroy Brain Tumor Cells in Patients (CARTREMENDOUS)	phase 1	GBM	EGFRvIII	10	ICV	University Of Oulu; Jyväskylä Central Hospital; Apollo Hospital	2020	Unknown
NCT06355908	IL13Rα2 CAR-T for Patients With r/​r Glioma	phase 1	Glioma	IL13Rα2	30	ICV	Beijing Tiantan Hospital	2024	Recruiting
NCT05540873	A Clinical Study of IL13Rα2 Targeted CAR-T in Patients With Malignant Glioma(MAGIC-I)	phase 1	Malignant Glioma	IL13Rα2	18	IV	National Cancer Center	2022	Recruiting
NCT04510051	CAR T Cells After Lymphodepletion for the Treatment of IL13Rα2 Positive Recurrent or Refractory Brain Tumors in Children	phase 1	Brain Neoplasm	IL13Rα2	18	ICV	City of Hope Medical Center	2020	Recruiting
NCT04003649	IL13Ra2-CAR T Cells With or Without Nivolumab and Ipilimumab in Treating Patients With GBM	phase 1	GBM	IL13Rα2	60	ICV/intracranital ICV	City of Hope Medical Center	2019	Recruiting
NCT04661384	Brain Tumor-Specific Immune Cells (IL13Ralpha2-CAR T Cells) for the Treatment of Leptomeningeal Glioblastoma, Ependymoma, or Medulloblastoma	phase 1	GBM; Ependymoma; medulloblastoma	IL13Rα2	30	ICV	City of Hope Medical Center	2021	Recruiting
NCT03696030	HER2-CAR T Cells in Treating Patients With Recurrent Brain or Leptomeningeal Metastases	phase 1	Metastatic Malignant Neoplasm in the Brain/Leptomeninges	HER2	39	ICV	City of Hope Medical Center	2018	Recruiting
NCT04903080	HER2-specific Chimeric Antigen Receptor (CAR) T Cells for Children With Ependymoma	phase 1	Ependymoma	HER2	50	IV	Pediatric Brain Tumor Consortium	2022	Recruiting
NCT05768880	Study of B7-H3, EGFR806, HER2, And IL13-Zetakine (Quad) CAR T Cell Locoregional Immunotherapy For Pediatric Diffuse Intrinsic Pontine Glioma, Diffuse Midline Glioma, And Recurrent Or Refractory Central Nervous System Tumors	phase 1	DIPG, DMG, Recurrent CNS Tumors	B7H3; EGFR806; HER2; IL13Rα2	72	ICV	Seattle Children’s Hospital	2023	Recruiting
NCT05544526	CAR T Cells to Target GD2 for DMG (CARMIGO)	phase 1	DMG	GD2	12	IV; ICV	University College, London	2023	Recruiting
NCT05298995	GD2-CAR T Cells for Pediatric Brain Tumours	phase 1	Brain Tumors	GD2	54	IV	Bambino Gesù Hospital and Research Institute	2023	Recruiting
NCT06221553	Safety and Efficacy of Loco-regional B7H3 IL-7Ra CAR T Cell in DIPG (CMD03DIPG)	phase 1	DIPG	B7H3	9	ICV	Chulalongkorn University	2024	Recruiting
NCT05835687	Loc3CAR: Locoregional Delivery of B7-H3-CAR T Cells for Pediatric Patients With Primary CNS Tumors	phase 1	Primary CNS Tumors	B7H3	36	ICV	St. Jude Children’s Research Hospital	2023	Recruiting
NCT05131763	NKG2D-based CAR T-cells Immunotherapy for Patient With r/​r NKG2DL + Solid Tumors	phase 1	GBM; Medulloblastoma; Hepatocellular carcinoma; Colon cancer	NKG2DL	3	IV	Fudan University	2021	Unknown
NCT04717999	Pilot Study of NKG2D CAR-T in Treating Patients With Recurrent Glioblastoma	NA	GBM	NKG2DL	20	ICV	UWELL Biopharma	2021	Unknown
NCT05353530	Phase I Study of IL-8 Receptor-modified CD70 CAR T Cell Therapy in CD70 + Adult Glioblastoma (IMPACT) (IMPACT)	phase 1	GBM	CD70	18	IV	University of Florida	2023	Recruiting
NCT05625594	Intracerebroventricular Administration of CD19-CAR T Cells (CD19CAR-CD28-CD3zeta-EGFRt-expressing Tcm-enriched T-lymphocytes) for the Treatment of Primary Central Nervous System Lymphoma	phase 1	CNSL	CD19	20	ICV	City of Hope Medical Center	2023	Recruiting
NCT03064269	CAR-T Therapy for Central Nervous System B-cell Acute Lymphocytic Leukemia	phase 1	CNS B-ALL	CD19	10	NA	Shanghai Unicar-Therapy Bio-medicine Technology	2017	Recruiting
NCT06213636	Fourth-gen CAR T Cells Targeting CD19/​CD22 for Highly Resistant B-cell Lymphoma/​Leukemia (PMBCL/​CNS-BCL). (BAH241)	phase 1/2	R/R Leukemia/Lymphoma patients with or without CNS	CD19; CD22	75	IV	Essen Biotech	2024	Recruiting
NCT04532203	A Study of CAR-T Cells Therapy for Patients With Relapsed and/​or Refractory Central Nervous System Hematological Malignancies	Early phase 1	R/​ R CNS hematological malignancies	CD19	72	IV	Zhejiang University	2020	Recruiting
NCT05651178	Human CD19-CD22 Targeted T Cells Injection for Refractory/​Relapsed Central Nervous System Leukemia/​Lymphoma Patients	Early phase 1	CNS Involvement of R/R B Cell malignancies	CD19; CD22	20	IV	Hrain Biotechnology Co., Ltd.	2022	Recruiting

*GBM *Glioblastoma, *IV *Intravenous, *ICV *Intracerebroventricular, *DIPG *Diffuse intrinsic pontine glioma, *DMG *Diffuse midline glioma, *CNS *Central nervous system, *CNSL *Ventral nervous system lymphoma, *ALL *Acute lymphoblastic leukaemia

### Gliomas

**Fig. 2:**
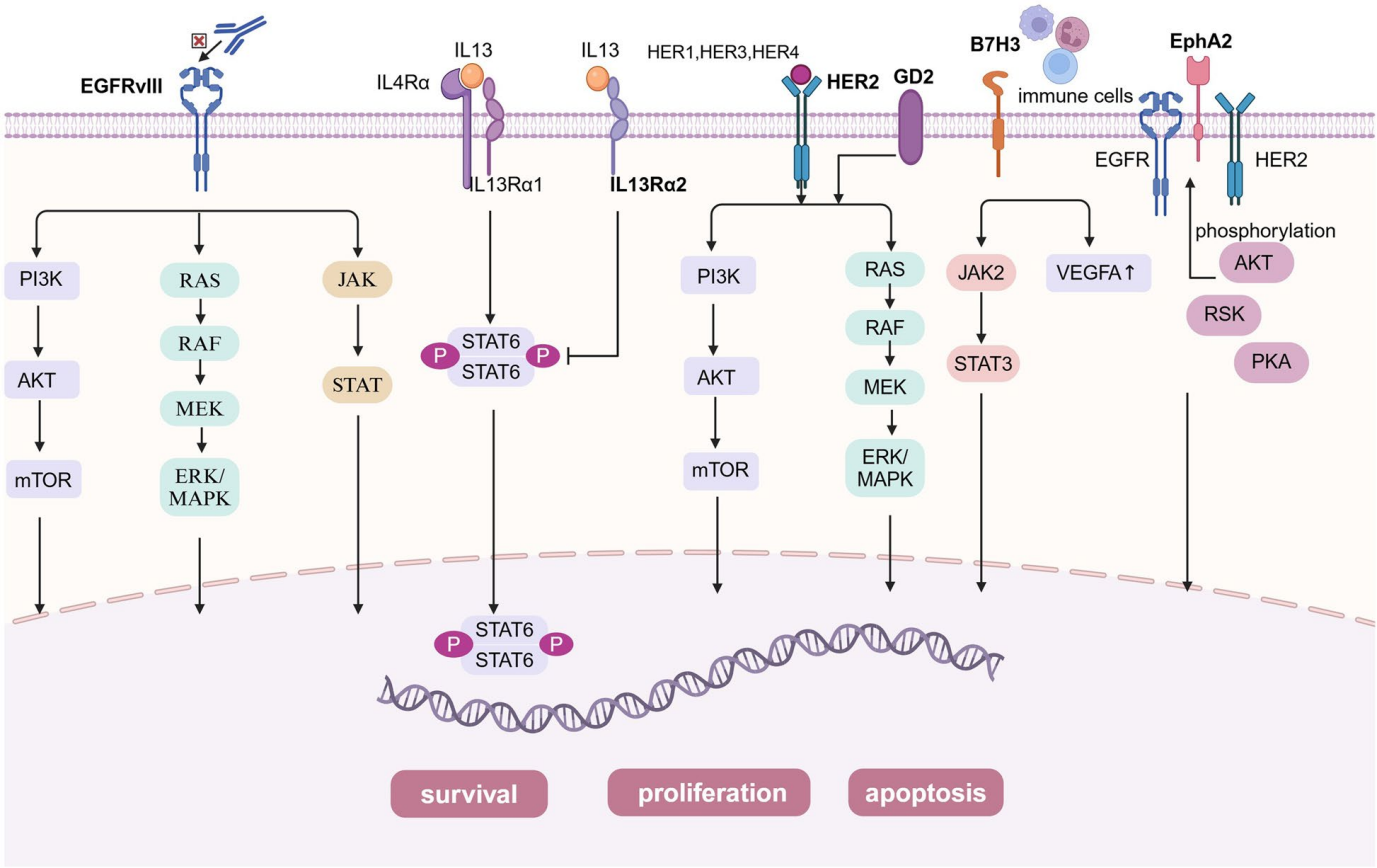
The mechanisms of action of clinically studied CAR-T targets in gliomas. EGFRvIII promotes GBM development mainly by enhancing the EGFRvIII-PI3K-AKT signalling pathway, EGFRvIII-Ras-Raf-MEK-ERK/MAPK signalling pathway and EGFRvIII-JAK-STAT signalling pathway; IL-13Rα2 competitively binds to IL-13, thereby inhibiting the STAT6 signaling pathway, promoting tumor cell invasion, metastasis, and proliferation, and inhibiting tumor cell apoptosis; Tumor formation via MAPK/ERK and PI3K/AKT/mTOR signalling pathways when HER2 gene expression is aberrant or GD2 is expressed; B7H3 enhanced cancer cell invasion by regulating the JAK2/STAT3 signalling pathway and upregulated the expression of vascular endothelial growth factor A (VEGFA) to promote tumor angiogenesis; Overexpressed EphA2 promotes tumor progression through AKT/RSK/PKA-mediated phosphorylation events

Malignant gliomas are the most common primary brain tumors within the CNS [57] and originate from various glial cell types, including astrocytes, oligodendrocytes, ependymal cells, and microglia [58]. Among these, GBM represents the most aggressive and lethal glioma, characterized by a high degree of heterogeneity. Despite the routine use of comprehensive treatments including surgical resection, radiotherapy, and chemotherapy, the prognosis for GBM remains dismal, with severely limited patient survival, and curative outcomes remain elusive with traditional therapies [59, 60]. Ependymomas constitute another significant glioma subtype, arising from ependymal cells lining the ventricular system. Approximately 50% of ependymomas in adults occur in the spinal cord, whereas nearly 90% are intracranial in pediatric patients. Notably, over half of pediatric patients with ependymomas experience recurrence despite standard treatments, and the cure rate for recurrent ependymomas remains low even after multiple surgeries, chemotherapy, and radiotherapy, resulting in poor clinical outcomes [61]. DMG and DIPG are common and significantly aggressive gliomas in children [62]. Research on CAR-T cell therapy for gliomas is rapidly advancing, with efforts focused on optimizing target selection and delivery strategies to enhance therapeutic efficacy and address current treatment limitations. We summarized the mechanisms of action of clinically studied CAR-T targets in gliomas (Fig. 2) and briefly analyzed the strengths and weaknesses of these targets and other targets that have only been explored in preclinical studies (Table 2).

**Table 2:** Analysis of the advantages and disadvantages of CAR-T therapy targets for glioma

Antigen	Characteristic	Antigen-expressing cancers	Advantages	Disadvantages
EGFRvIII [44]	an 801 bp in-frame deletion of exons 2 to 7	glioblastoma; lung cancer; breast cancer; ovarian cancer; prostate cancer;	• specifically expressed in tumors	• after CAR-T infusion, tumor samples showed increased levels of immunosuppressive molecules: IDO1,FOXP3,IL-10,PD-L1,and TGFβ.
IL13Rα2 [63, 64]	NA	the central nervous system; melanoma; lung cancer; prostate cancer	• aside from high testicular expression, it shows no expression elsewhere in the human body.	• on-target, off-tumor toxicity
HER2 [65–67]	a member of the EGFR family	glioblastoma; ependymom; medulloblastoma; CNS cancer stem cells; breast cancer; renal cell carcinoma; lung adenocarcinoma	• expressed across a spectrum of biologically diverse CNS tumors	• the lower HER2 expression in CNS tumors relative to breast cancer, limit the application of HER2-targeting antibodies against CNS tumors; • on-target, off-tumor toxicity
GD2 [68, 69]	b-series ganglioside	glioblastoma; astrocytoma; retinoblastoma; Ewing’s sarcoma; Rhabdomyosarcoma; osteosarcoma; leiomyosarcoma; liposarcoma; fibrosarcoma; small cell lung cancer; melanoma; breast cancer	• tumor cells show high-density expression, while normal cells exhibit limited expression.	• on-target, off-tumor toxicity; • GD2 is also expressed in low amounts on peripheral nerves and brain parenchyma
B7H3 [70, 71]	316 amino acids encoded by 12 exons located in chromosome 9 in murine while in 15q24.1 in humans	Glioblastoma; pancreatic ductal adenocarcinoma; ovarian cancer; neuroblastoma; diffuse intrinsic pontine glioma; medulloblastoma; craniopharyngiom; atypical teratoid/rhabdoid tumor; metastatic brain tumors	• expressed on tumor cells and abnormal vessels, but rarely on normal cells.	• on-target, off-tumor toxicity
EphA2 [72]	a member of the Eph family of receptor tyrosine kinases (RTK)	breast cancer; lung cancer; glioblastoma; melanoma	• expressed highly in glioblastoma but only at low levels in normal brain tissue	• on-target, off-tumor toxicity
P32 [57, 73]	a mitochondrial protein	breast cancer; glioblastoma; prostate cancer; melanoma cancer; lung cancer; pancreatic cancer; colon cancer; malignant pleural mesothelioma	• expressed glioma cells and tumor derived endothelial cells	• on-target, off-tumor toxicity
CSPG4 [74, 75]	a cell surface type I transmembrane protein	melanoma; triple-negative breast cancer; glioblastoma; mesothelioma; sarcoma	• expressed on tumor cells, but rarely on normal cells.	• on-target, off-tumor toxicity
NKG2DL [76–78]	an activating receptor	multiple myeloma; ovarian carcinoma; lymphoma; osteosarcoma	• expressed on tumor cells and cancer stem cells, but rarely on normal cells.	• on-target, off-tumor toxicity
CD70 [79, 80]	a type II transmembrane protein	renal cell carcinoma; leukemia; non-small cell lung cancer; melanoma; glioblastoma	• expressed on tumor cells, but rarely on normal cells.	• on-target, off-tumor toxicity
CD133 [81, 82]	transmembrane glycoprotein	hepatocellular carcinoma; glioblastoma; pancreatic cancer; gastric cancer; intrahepatic cholangiocarcinomas	• expressed by cancer stem cells of various epithelial cell origins	• on-target, off-tumor toxicity

#### EGFRvIII and EGFR

EGFRvIII is a common tumor-specific mutation widely expressed in GBM and other tumors but rarely expressed in normal tissues [83, 84]. Its expression results from an 801 bp in-frame deletion of exons 2 to 7, resulting in a new glycine residue. This leads to the absence of the ligand-binding domain and low-level constitutive activity of EGFRvIII. This alteration confers tumor specificity, immunogenicity, and oncogenicity to the extracellular domain of EGFRvIII [85]. EGFRvIII establishes a regulatory network of signaling pathways through ligand-independent autophosphorylation and tyrosine kinase activity, which plays a significant role in GBM growth, metastasis, and angiogenesis. Specifically, EGFRvIII enhances the EGFRvIII-PI3K-AKT signaling pathway, the EGFRvIII-Ras-Raf-MEK-ERK/MAPK signaling pathway, and the EGFRvIII-JAK-STAT signaling pathway to promote the proliferation, survival, invasion, and angiogenic capabilities of GBM [86]. Therefore, EGFRvIII has become an important target for CAR-T therapy owing to these characteristics.

EGFRvIII CAR-T therapy has demonstrated efficacy in GBM murine models; however, its effectiveness is frequently constrained by the tumor’s immunosuppressive microenvironment, limiting the ability to fully control large gliomas. Studies have shown that IL-12 enhances the cytotoxicity of EGFRvIII CAR-T cells and remodels the immune-TME by reducing the proportion of Treg cells and increasing pro-inflammatory CD4 + T cells [87]. These findings suggest that combining IL-12 with EGFRvIII CAR-T therapy may enhance treatment efficacy for GBM. Moreover, inhibition of vascular endothelial growth factor (VEGF) can aid in improving the infiltration and distribution of EGFRvIII CAR-T cells within GBM’s immunosuppressive microenvironment, thereby suppressing tumor growth and extending the survival time of mice [88]. These strategies indicate that the efficacy of EGFRvIII CAR-T cells in treating GBM can be improved by combining immunomodulatory factors and anti-angiogenic agents. However, additional clinical studies are necessary to confirm the efficacy and safety of these approaches and identify the optimal therapeutic regimen to improve clinical outcomes.

Johnson et al. developed a humanized CAR-T cell targeting EGFRvIII, demonstrating its ability to eradicate EGFRvIII-positive GBM in subcutaneous and orthotopic xenograft models [89]. Based on these results, their research center initiated a phase I clinical trial in which 10 patients with recurrent GBM (rGBM) received EGFRvIII CAR-T cell therapy, with a median survival of approximately eight months. One patient showed no disease progression for over 18 months following a single infusion of EGFRvIII CAR-T cells. Among these patients, the incidence of neurological events was 30%, with no cases of CRS or off-tumor toxicity targeting EGFR. Seven patients underwent surgery either within two weeks or two months post-infusion. Tumor specimens showed higher EGFRvIII CAR-T cell concentrations in the brain compared to peripheral blood at two weeks post-infusion but lower levels in the brain than the blood at two months post-infusion. This indicates that EGFRvIII CAR-T cells effectively trafficked to active GBM regions and possibly proliferated in situ, albeit transiently. The study also found that after EGFRvIII CAR-T cell infusion, the expression of immunosuppressive molecules such as indoleamine 2,3-dioxygenase 1 (IDO1) and FoxP3 were upregulated, and there was a notable increase in Tregs. These factors collectively initiated an adaptive immune escape mechanism, thereby diminishing the antitumor effects [44]. Despite the poor prognosis of the patients, this study demonstrated the feasibility and safety of manufacturing and infusing EGFRvIII CAR-T cells. Nonetheless, the utilization of EGFRvIII CAR-T cells did not yield clinically significant results in patients with GBM. This study reported that almost all patients experienced transient hematologic toxicity, and two suffered from severe hypoxia. The median progression-free survival (PFS) was only 1.3 months, and the median overall survival (OS) was 6.9 months [90]. This suggests the need for further optimization of treatment regimens and additional research into the long-term efficacy and safety of EGFRvIII CAR-T cells to optimize future treatment strategies.

Although EGFRvIII demonstrates promising targeting capability and therapeutic potential in gliomas, the widespread expression of EGFR and its crucial role in tumorigenesis render it an equally significant target antigen. As a member of the ErbB/HER receptor family, EGFR is a transmembrane glycoprotein consisting of 1,186 amino acids, commonly found in various human cancers, including GBM, non-small cell lung cancer, and breast cancer, where it is frequently overexpressed and activated. However, the ubiquitous presence of EGFR in normal tissues poses a substantial challenge [91], as therapies targeting EGFR may lead to significant off-tumor toxicity [92]. To address this challenge, Dobersberger M et al. developed a CAR-T cell engineering strategy that specifically recognizes the conformational changes in EGFR following ligand activation. This approach enables CAR-T cells to effectively distinguish between tumor cells and unactivated EGFR present in normal tissues, thereby reducing toxicity to normal tissues and enhancing therapeutic specificity for EGFR-positive solid tumors [93]. In constructing a CAR targeting EGFR expression in CNS tumors, researchers selected the single-chain variable fragment (scFv) derived from mAb806. This choice is based on mAb806’s ability to bind both to EGFRvIII and to full-length EGFR expressed due to gene amplification while also exhibiting tumor specificity. This specificity is primarily attributed to the conformational changes in EGFR resulting from overexpression in tumor cells, which expose the binding site of mAb806; conversely, the normal conformation of EGFR in normal cells impedes this binding [94].Through this strategy, EGFR806-CAR T cells exhibited significant anti-tumor activity in mouse models [95]. Moreover, CART.BiTE cell therapy employs a bicistronic structure to co-express both an EGFRvIII-specific CAR and an EGFR-specific BiTE. This approach facilitates the direct targeting of EGFRvIII-positive tumor cells while also recruiting bystander T cells to attack EGFR-positive but EGFRvIII-negative tumor cells, thereby overcoming antigen heterogeneity and reducing toxicity to normal tissues [96].

In summary, while CAR T-cell therapies targeting EGFR and EGFRvIII exhibit significant potential and therapeutic specificity in targeting tumor cells, the widespread expression of EGFR continues to pose a risk of unintended toxicity to normal tissues, highlighting the necessity for further research to optimize treatment strategies that balance efficacy and safety.

#### IL13Rα2

IL-13Rα2 is expressed in most patients with diffuse high-grade glioma (HGG) but not in normal brain tissue [64] and it is associated with poor tumor prognosis [97, 98]. IL-13Rα2 is a decoy receptor for IL-13, with a higher affinity for IL-13 than IL-13Rα1. Under normal conditions, the IL-13/IL-13Rα complex binds to IL-4Rα to form an IL-13/IL-13Rα/IL-4Rα complex, which activates STAT6 through its intracellular tail. STAT6 subsequently translocates to the nucleus to regulate gene transcription, promoting apoptosis. However, in GBM, IL-13Rα2 competitively binds to IL-13, thereby inhibiting the STAT6 signaling pathway, promoting tumor cell invasion, metastasis, and proliferation, and inhibiting tumor cell apoptosis [64, 99]. Therefore, IL-13Rα2 is considered a tumor marker specifically expressed in various tumors owing to these characteristics.

Previous researchers screened and identified a scFv clone termed 14−1, which exhibits approximately five times higher binding affinity for IL-13Rα2 compared to the previous clone 4−1. The study demonstrated that these scFv-IL-13Rα2-CAR-T cells exhibited significant antitumor activity in vitro and in vivo, effectively killing IL-13Rα2-positive tumor cells while showing low toxicity in non-tumor-bearing mice [100]. These findings indicate that scFv-IL-13Rα2-CAR-T therapy is a potentially effective antitumor treatment modality, warranting further preclinical and clinical investigations.

IL-13Rα2 CAR-T cells were locally injected into resected tumor cavities of three patients with rGBM. Two patients showed evidence of a transient antitumor response. Magnetic resonance imaging (MRI) of one patient post-treatment showed increased necrotic tumor tissue volume at the injection site, and all patients tolerated the treatment well [101]. The largest clinical trial to date for CAR-T cell therapy targeting solid tumors utilized a localized delivery method targeting IL-13Rα2 to treat rGBM and other HGGs. The results indicated that among the 58 patients who received the treatment, 29 (50%) achieved stable disease (SD) or improvement. The median OS for all patients was eight months, with the rGBM subgroup achieving a median OS of 7.7 months. Two cases of partial remission (PR) and one of complete remission(CR) were observed with additional CAR-T treatment cycles. However, 35% of the patients experienced grade 3 or higher toxicities, including one case of grade 3 encephalopathy and one of ataxia [102]. In summary, localized IL-13Rα2-targeted CAR-T therapy demonstrated safety and efficacy in a subset of patients, however, some patients experienced toxic reactions, underscoring the necessity for diligent monitoring and management of potential adverse effects in clinical applications.

Currently, autologous CAR-T cells are widely used; however, their application is limited. Therefore, researchers have developed allogeneic IL-13Rα2 CAR-T cell products for GBM treatment. They employed zinc finger nucleases (ZFN) to genetically engineer CAR-T cells, knocking out the glucocorticoid receptor and resulting in modified CAR-T cells termed GRm13Z40-2 that are resistant to glucocorticoids. GRm13Z40-2 and aldesleukin were intracranially injected into six patients with GBM, in addition to systemic dexamethasone maintenance therapy. Among the six treated patients, four showed signs of transient tumor reduction and/or necrosis at the T-cell injection site [103]. This treatment demonstrated efficacy in certain patients, suggesting that this therapy may constitute an effective strategy for GBM treatment.

#### HER2

Human epidermal growth factor receptor 2 (HER2) is a tyrosine kinase receptor membrane [104] glycoprotein encoded by the ErbB gene on chromosome 17q21. This gene is classified as a proto-oncogene and typically plays a role in regulating cell growth and division. However, HER2 gene expression dysregulation increases the susceptibility of normal cells and tissues to malignant transformation, leading to tumorigenesis [105]. This process is primarily mediated through the MAPK/ERK and PI3K/AKT/mTOR signaling pathways [106]. HER2 is expressed in approximately 80% of patients with GBM, whereas its expression in normal neural tissue is significantly restricted [107, 108]. Overexpression of the protein encoded by this gene enhances tumor aggressiveness, resulting in poor prognosis and contributing to drug resistance development [65, 109]. Considering its high expression in GBM and its correlation with tumor progression, HER2 is regarded as a critical target for CAR-T therapy, potentially offering novel therapeutic strategies for GBM treatment.

HER2-specific CAR-T cells have been shown to exhibit high selectivity for HER2-positive GBM. One study co-cultured third-generation HER2 CAR-T cells, which contain CD28 and CD137 costimulatory domains, with HER2-positive U251 GBM cells. This study demonstrated that HER2 CAR-T cells exhibited significant cytotoxicity against the tumor cells, accompanied by a significant increase in the secretion levels of IL-2 and IFN-γ [65]. HER2 CAR-T cells exhibited significant anti-tumor activity and robust immune responses in preclinical studies, thereby reinforcing their potential utility for future clinical applications.

A phase 1 dose-escalation clinical study used second-generation HER2-CAR-T (FRP5.CD28.ζ) cells derived from virus-specific T cells (VST), reporting that 17 patients who received CAR-T cell infusions exhibited good tolerability. Among them, eight patients showed signs of PR or SD, with a median OS of 11.1 months for all patients. These findings suggest that HER2-CAR VST infusion is both safe and feasible in clinical applications, demonstrating notable efficacy in treating adult GBM. However, although these cells remained detectable post-infusion, no significant expansion of CAR-T cells was observed in peripheral blood, suggesting a limited in vivo response. Moreover, the assessment of CAR-T therapy in pediatric CNS tumors remains limited, reflecting further constraints on in vivo responses [66]. In a previous study, autologous CD4 + and CD8 + T cells expressing HER2-specific CAR were transduced using a lentivirus and delivered locally via intratumoral or intraventricular administration. Among the three patients who received this treatment, no dose-limiting toxicity occurred, and evidence of CNS immune activation was observed, as indicated by elevated levels of CXCL10 and CCL2 in cerebrospinal fluid and serum, indicating that the treatment was well tolerated [110]. This study provides preliminary evidence for the feasibility of repeated administration of HER2-specific CAR-T cells for CNS tumor treatment in pediatric and young adult populations.

#### GD2

GD2 is a disialoganglioside prominently expressed in several malignancies, such as neuroblastoma, GBM, and melanoma. GD2 expression in normal tissues is primarily limited to specific cells within the central and peripheral nervous systems, exhibiting relatively low expression levels [111]. GD2 inhibits T cell proliferation, induces apoptosis, and suppresses human CD34 + cell differentiation into mature dendritic cells [68, 112]. Furthermore, GD2 plays a role in tumor cell invasion, angiogenesis, and metastasis [113]. Therefore, GD2 has become a highly attractive tumor target and an excellent candidate for cancer immunotherapy owing to these properties.

A mouse glioma xenograft model showed that mice that received intravenous injections of GD2 CAR-T cells exhibited a longer average survival time of 47 days compared to control and T cell groups, indicating that intravenously administered GD2 CAR-T cells were able to penetrate the brain tumor tissue. Additionally, the research team developed a CAR-T cell capable of secreting IL-15, resulting in more comprehensive and sustained tumor control [111]. However, another study demonstrated that while intratumoral injection of GD2 CAR-T cells was effective, intravenous administration was not, which can be attributed to the lack of cross-reactivity with GD2 in mice [114]. Further studies are required to determine the optimal administration route for GD2 CAR-T cells. Additionally, studies have shown that radiation therapy can enhance the infiltration and expansion of GD2 CAR-T cells within the TME, thereby boosting the antitumor immune response [115]. This suggests that combining radiotherapy with CAR-T cell therapy may be a promising strategy for enhancing the treatment efficacy of malignant tumors, necessitating further investigation in subsequent studies. DMG with H3K27M mutations is a fatal CNS tumor in children [116]. Preclinical studies have demonstrated that GD2 CAR-T cells can significantly eliminate tumors in patient-derived H3K27M + DMG orthotopic xenograft models [117], offering new hope for the treatment of these deadly childhood cancers.

Researchers have developed and applied fourth-generation CAR-T cells (4S CAR) incorporating CD28 transmembrane and cytoplasmic regions, intracellular TRAF-binding domain of the co-stimulatory molecule 4-1BB, intracellular structural domain of the CD3z chain, and suicide-inducing caspase 9 gene. Overall, eight patients with GD2-positive GBM were enrolled in this study. All patients underwent lymphodepletion prior to CAR-T cell administration, with CAR-T cells delivered either via intravenous infusion alone or in combination with intracavitary injection. The results demonstrated that 4S CAR-T cells persisted at a low copy number in peripheral blood for 1–3 weeks following expansion. Moreover, 50% of these patients (4/8) experienced PR within 3–24 months post-infusion, 37.5% (3/8) exhibited progressive disease (PD) within 6–23 months post-infusion, and 12.5% (1/8) had stable disease at four months post-infusion. The median OS for the overall patient cohort was 10 months, and no serious adverse events were reported [118]. These findings suggest that 4S CAR-T cells possess a favorable safety and tolerability profile for the treatment of patients with GD2-positive GBM. However, the OS rate remains limited and warrants further investigation with an expanded sample size. Supported by preclinical studies, GD2-CAR-T therapy was administered to patients with H3K27M-mutant DIPG and DMG of the spinal cord. Among the four enrolled patients, three exhibited clinical and radiographic improvements, in addition to increased levels of pro-inflammatory cytokines in the blood and cerebrospinal fluid, suggesting effective interaction between CAR-T cells and tumor cells. Patients experienced CNS symptoms and signs related to CAR-T therapy; nevertheless, these toxic effects were reversible with appropriate management strategies [119]. Overall, these findings highlight the considerable potential of GD2-CAR-T therapy in enhancing the clinical outcomes of patients with DIPG and DMG. A previous study developed C7R-GD2 CAR-T cells expressing the IL-7 receptor (IL-7R) to enhance GD2 CAR-T cell efficacy in treating CNS tumors. This modification enabled C7R-GD2 CAR-T cells to activate downstream signaling pathways independently of IL-7, thereby enhancing the effectiveness of CAR-T therapy. Among the 11 patients enrolled in the study, eight were diagnosed with H3K27-mutant DMG. Patients receiving GD2 CAR-T treatment exhibited neurofunctional improvement for less than three weeks, with no cases of CRS or tumor inflammation-associated neurotoxicity (TIAN). TIAN represents a form of neuronal dysfunction resulting from localized inflammation and the consequent transient edema, which contrasts with the generalized and diffuse cerebral edema seen in ICANS. TIAN can be categorized into two types: Type 1 primarily manifests as inflammatory edema, resulting in elevated intracranial pressure and limited mechanical spaces; Type 2 indicates local inflammation triggered by immunotherapy, subsequently leading to functional impairment in specific neural regions [120]. In contrast, patients treated with C7R-GD2 CAR-T cells experienced a median duration of neurofunctional improvement of five months, with 88% achieving PR or SD. Additionally, the PFS of patients who received C7R-GD2 CAR-T cells was significantly longer than that of those who received GD2 CAR-T cells [121]. These results suggest that C7R-GD2 CAR-T therapy exhibits excellent clinical efficacy in patients with CNS tumors.

#### B7H3

B7H3, also referred to as CD276, is a highly conserved type I transmembrane protein encoded by human chromosome 15, comprising 316 amino acids [122, 123]. B7H3 exists in two human isoforms owing to variations in its extracellular domain: 2IgB7-H3 and 4IgB7-H3 [70]. B7H3 is highly expressed in a variety of tumor cells and within TMEs, particularly during pathological angiogenesis. B7H3 expression is markedly elevated in GBM, neuroblastoma, and ovarian cancer [71, 124], where it is strongly associated with tumor malignancy and poor prognosis [125]. B7H3 is expressed in certain peritumoral tissues; however, its expression remains low and is either minimally detectable or nearly absent in normal tissues and organs [126]. It enhances cancer cell invasiveness by regulating the JAK2/STAT3 signaling pathway and facilitates angiogenesis in the TME by upregulating vascular endothelial growth factor A (VEGFA) expression [70]. Therefore, B7H3 has been identified as a promising therapeutic target for the selective disruption of tumors and their vascular networks.

A third-generation B7H3 CAR-T cell was engineered, and its potent cytotoxicity on primary GBM cells and GBM cell lines was demonstrated through in vitro assays. The median survival in the B7H3 CAR-T cell-treated group was significantly prolonged compared to the control group in xenograft models, However, tumor recurrence was observed in the brains of mice receiving CAR-T cell therapy, suggesting that this may be due to insufficient CAR-T cell dosing [125]. Therefore, it is crucial to pay special attention to the dosing of CAR-T cells to optimize therapeutic outcomes. Similarly, another study demonstrated that B7H3 is overexpressed in GBM specimens, and CAR-T cells targeting B7H3 can effectively inhibit tumor cell proliferation [127]. These studies demonstrated that B7H3 CAR-T cells exhibit significant anti-tumor effects in GBM treatment; nevertheless, a potential to further enhance therapeutic efficacy remains. Recent research indicates that pre-treatment with radiation before B7H3 CAR-T cell infusion can significantly improve therapeutic efficacy in solid tumor models [128]. Moreover, oncolytic adenoviruses carrying CXCL11 can enhance the efficacy of B7H3 CAR-T cells in GBM treatment by remodeling the immunosuppressive microenvironment [129]. Researchers have developed a novel B7H3 CAR-T cell incorporating the transmembrane and immunoglobulin domain–containing 2 (TMIGD2) co-stimulatory domain, which is a co-stimulatory factor for T cells and NK cells, to further optimize CAR-T therapy. B7H3 CAR-T cells with the TMIGD2 co-stimulatory domain exhibited superior anti-tumor activity, enhanced expansion, and improved persistence compared to traditional CAR-T cells incorporating CD28 and/or 4-1BB co-stimulatory domains. The underlying mechanisms include maintenance of mitochondrial metabolism, reduced cytokine production, decreased cell exhaustion, and an increased proportion of central memory and CD8 + T cells [130]. This CAR-T cell type exhibits notable advantages in combating solid tumors, thereby warranting further research and development.

Intracranial administration of B7H3 CAR-T cells in patients with DIPG has shown good tolerance, with B7H3 CAR-T cells persisting in the cerebrospinal fluid, showing evidence of immune activation. Notably, one patient exhibited continuous clinical and radiological improvement over a 12-month follow-up period, without experiencing dose-limiting toxicity [131]. Vitanza et al. reported data from a clinical trial involving 21 pediatric patients with DMG who received B7H3 CAR-T cell therapy. Meanwhile, Mahdi et al. presented results from a clinical trial at Stanford University, in which nine patients with DMG were treated with B7H3 CAR-T cells. Of these, four (44%) experienced tumor reduction of more than 50%, and one achieved CR [132]. These findings suggest that B7H3 CAR-T cells have potential in glioma treatment, as evidenced by significant tumor shrinkage and complete remission in some patients.

#### EphA2

EphA2 is a tyrosine kinase receptor that binds ligands of the EphrinA family [133]. It is highly expressed in tumors such as GBM, breast cancer, lung cancer, and melanoma, whereas its expression is limited in healthy tissues [72]. Elevated EphA2 expression correlates with poor prognosis, reduced survival, and increased metastasis rates in patients with tumors. In cancer cells, EphA2 exhibits a dual role: its ligand-dependent function inhibits cancer cell invasion and migration upon ligand binding, whereas its ligand-independent kinase activity involves overexpressed EphA2 altering downstream signaling pathways through dimerization with E-cadherin, EGFR, HER2, and integrins. Additionally, EphA2 can be activated through phosphorylation events mediated by AKT/RSK/PKA [72, 134], thereby promoting tumor progression.

CAR-T cells targeting EphA2 have shown significant antitumor effects in preclinical studies [135]. A research team developed two third-generation EphA2 CAR-T cells: EphA2-a CAR-T and EphA2-b CAR-T. In vitro experiments demonstrated that both CAR-T cells could be activated by EphA2-positive tumor cells, with EphA2-a CAR-T exhibiting significantly higher tumor-killing efficiency compared to EphA2-b CAR-T. In vivo experiments similarly demonstrated that both CAR-T cells significantly prolonged mouse survival, which may be attributed to the modulation of the CXCR-1/2 signaling pathways and moderate increases in IFN-γ levels. However, the reduced efficacy of EphA2-b CAR-T may attributed to excessive IFN-γ expression, which leads to PD-L1 upregulation in GBM cells and consequently diminishes the antitumor effect [136]. These findings indicate that EphA2 CAR-T cell therapy has the potential for GBM treatment.

A clinical trial of three patients with rGBM showed that EphA2 CAR-T therapy resulted in SD in one patient and PD in two patients, with OS ranging from 86–181 days. Two patients developed grade 2 CRS accompanied by pulmonary edema, which resolved after dexamethasone treatment, indicating favorable tolerability following EphA2 CAR-T infusion. However, the therapeutic effect was suboptimal, and its duration was relatively brief, with the occurrence of pulmonary edema suggesting potential on-target off-tumor toxicity [137]. Consequently, further optimization is required to improve its safety and durability, as well as to broaden its potential for clinical application.

#### P32

P32, also known as gC1qR/HABP/C1qBP, is a mitochondrial protein that acts as a receptor for CGKPK and is expressed on surfaces of tumor cells and endothelial cells involved in tumor angiogenesis [73, 138]. Rousso Noori et al. demonstrated that P32 is specifically expressed in GBM cells, making it a promising target for CAR-T therapy. Further studies showed that P32 CAR-T cells can specifically recognize and eliminate P32-expressing glioma cells and tumor-derived endothelial cells in vitro and significantly inhibit tumor growth in xenograft and syngeneic mouse models [57]. These findings indicate that P32 CAR-T cells exhibit both antitumor activity and anti-angiogenic effects. Therefore, P32 CAR-T cells represent a promising therapeutic option for patients with GBM, although no related clinical trials have been reported to date.

#### CSPG4

Chondroitin sulfate proteoglycan 4 (CSPG4) is a type I transmembrane protein widely expressed across multiple malignant tumors, including melanoma, triple-negative breast cancer, mesothelioma, and sarcoma [74]. Notably, CSPG4 is expressed in up to 67% of GBM, whereas its expression is more limited in normal tissues, and it is strongly correlated with reduced patient survival [75, 139, 140]. CSPG4 CAR-T cells exhibited potent inhibitory effects on a glioblastoma neurosphere (GBM-NS) model in vitro and in vivo. In GBM-NS models with low CSPG4 expression, microglial cells surrounding the tumor induced CSPG4 upregulation on the tumor cell surface by releasing TNF-α, thereby enhancing the therapeutic efficacy of CAR-T cells [139]. Preclinical studies demonstrated significant efficacy of CSPG4 CAR-T in GBM treatment; however, further clinical trial data is required to validate its efficacy and safety.

#### NKG2DL

Natural killer cell group 2 member D (NKG2D) is an activating cell surface receptor [76] primarily expressed on immune cells, including NK cells and CD8 + T cells. Its ligands (NKG2DLs) consist of eight distinct proteins in humans, including major histocompatibility complex (MHC) class I chain-related molecules (MICA, MICB) and six UL16-binding proteins (ULBPs) [141]. These ligands are widely expressed on the surface of GBM cells, cancer stem cells, and other tumor cells [77]. Preclinical studies reveal that murine NKG2D CAR-T cells demonstrate strong cytolytic activity against glioma cells in vitro, significantly prolonging survival in a glioma mouse model, with additional long-term protective effects. Moreover, local radiotherapy enhanced the migration of NKG2D CAR-T cells to the tumor site, thereby enhancing their cytotoxic efficacy [142]. Meister et al. developed mRNA-based CAR-T cells co-expressing the NKG2D receptor and pro-inflammatory cytokines IL-12 and IFNα2, which efficiently killed mouse glioma cell lines in vitro and exhibited anti-tumor activity in a glioma mouse model with intravenous and intratumoral administration [143]. Further studies demonstrated that human CAR-T cells expressing NKG2D could target GBM and cancer stem cells, efficiently lysing these cells [78]. NKG2D CAR-T cells have shown significant efficacy against gliomas in preclinical studies; however, no clinical trial data have been reported to date, necessitating further clinical validation of these findings.

#### CD70

CD70, a member of the tumor necrosis factor superfamily and ligand for CD27, is overexpressed in renal cell carcinoma, leukemia, non-small cell lung cancer, melanoma, and GBM [45, 144], where its high expression is strongly correlated with reduced patient survival [145]. The mechanisms underlying this association may involve the induction of apoptosis in CD8+ T cells, recruitment of TAMs to the GBM microenvironment resulting in immunosuppression, and participation in glioma chemokine production [79, 145]. CD70 is transiently expressed in activated T cells, B cells, and mature dendritic cells, with very low expression levels in most normal tissues. Notably, CD70 expression is significantly elevated in samples from patients with recurrent GBM compared to primary GBM.

Human- and murine-derived CD70 CAR-T cells demonstrated tumor regression in in vitro studies, human xenograft models, and syngeneic in situ glioma models. Researchers developed CD70 CAR-T cells modified with the rabies virus glycoprotein-derived RVG29 peptide (70R CAR-T) to address the challenge of CD70 CAR-T cells penetrating the BBB. In vitro cellular experiments demonstrated that 70R CAR-T cells exhibited markedly increased cytotoxic activity against CD70-positive glioma cells. Furthermore, 70R CAR-T cells in vivo demonstrated improved capability in crossing the BBB and enhanced therapeutic potency compared to conventional CD70 CAR-T cells. However, it is crucial to acknowledge that the RVG29 peptide, being an exogenous substance, may be identified by the host immune system as “non-self” potentially inducing both humoral and cellular anti-CAR immunity, which could consequently restrict its overall efficacy and influence the persistence of CAR-T cells. Despite these concerns, the study provided preliminary insights into the enhanced killing mechanisms of 70R CAR-T cells, which exhibited a lower apoptosis rate, increased proportion of central memory T cells (TCM), and decreased proportion of effector memory T cells (TEM), leading to improved phenotypic characteristics [80]. Nevertheless, the generalizability of these research findings is constrained and necessitates further validation. Therefore, future research should investigate the immunogenicity of the RVG29 peptide more comprehensively and evaluate its safety and tolerability across diverse patient populations to ensure that the clinical application of 70R CAR-T cells is not impeded by immunogenicity concerns.

#### CD133

CD133 is a transmembrane glycoprotein widely recognized as a crucial marker of malignant tumor recurrence and poor prognosis, and it is expressed in a variety of malignant tumors, including hepatocellular carcinoma, pancreatic cancer, and gastric cancer [81]. In addition, CD133 is also a marker for tumor stem cells (CSCs) and endothelial progenitor cells (EPCs) [146]. In GBM, CD133 expression is significantly elevated compared to low-grade gliomas, and studies have shown that GBM patients with high CD133 expression have a poorer prognosis [147]. In a humanized mouse model of GBM, CD133 CAR-T cells demonstrated robust anti-tumor activity and significant therapeutic efficacy while not triggering acute systemic toxicity by intratumoral injection [147]. However, data from clinical trials targeting CD133 CAR-T cells for the treatment of GBM remain unpublished.

#### multi-targeted CAR-T

Given the role of TGF-β in the immunosuppressive microenvironment, Chang ZL, et al. designed CAR-T cells to simultaneously target GBM and TGF-β within the TME. These CAR-T cells directly kill tumor cells by targeting IL-13Rα2 and convert TGF-β from an immunosuppressant to an immunostimulant through TGF-β targeting [148]. Compared to traditional IL-13Rα2-targeting CAR-T cells, the IL-13Rα2/TGF-β CAR-T cells demonstrate enhanced efficacy against GBM in mouse models and possess the ability to resist and remodel the immunosuppressive microenvironment [149]. What’s more, the research team has developed a dual-target CAR-T cell therapy for GBM, named CART-EGFR-IL3Rα2 cell therapy. This therapy administers CAR-T cells to the cerebrospinal fluid via intrathecal injection. Results indicated that in the six patients who received the dual-target CAR-T cell therapy, MRI scans showed a reduction in brain tumor size, with some patients maintaining this reduction for several months [150]. This research offers new insights and methods for GBM immunotherapy, potentially improving patient prognosis. However, further large-scale clinical trials are necessary to evaluate the long-term safety and efficacy of this therapy.

Although EGFRvIII is highly specific, it exhibits heterogeneous expression [89]. CAR-T cells that selectively target this antigen can allow antigen-negative tumor cells to escape [151], leading to insufficient efficacy. Some antigens, such as EphA2 and IL13Rα2, are uniformly expressed in GBM cells; however, they have specificity drawbacks as they are also expressed in some healthy tissues, such as the liver, kidneys, and esophagus [152]. Targeting such antigens with CAR-T cells poses the potential risk of attacking normal tissue cells. To address this challenge, researchers have designed SynNotch-CAR T cells that recognize multiple antigen combinations. The mechanism involves first activating the expression of the CAR through a synNotch receptor, which recognizes the tumor-specific but heterogeneously expressed EGFRvIII antigen. Upon recognition, the engaged CAR undergoes cleavage, leading to the transcriptional upregulation of a second CAR. This second CAR is responsible for killing cancer cells by recognizing antigens such as EphA2 or IL13Rα2, which are uniformly expressed in cancer cells but not tumor-specific [153, 154]. In a xenogeneic GBM mouse model, the infusion of synNotch-CAR T cells demonstrated significantly higher antitumor efficacy and CAR-T cell persistence compared to traditional constitutively expressed CAR-T cells. Additionally, a higher proportion of CAR-T cells remained in the naïve/stem cell memory state, thereby reducing their exhaustion levels [154]. In addition, SynNotch-CAR T cells have effectively addressed the limitations of traditional CAR-T therapy by employing a multi-antigen recognition strategy. They have demonstrated enhanced therapeutic efficacy and improved safety profiles, offering novel approaches for GBM treatment. However, one potential limitation of the SynNotch-CAR strategy is the risk of on-target off-tumor activity. If T cells expressing the second CAR leave the TME, they could potentially target normal tissues expressing EphA2 or IL13Rα2, leading to off-target effects. This issue underscores the necessity for a careful balance between antitumor efficacy and safety. To enhance the specificity and safety of CAR-T cell therapy, the research team developed two CARs that target distinct tumor antigens. This strategy’s core involves utilizing distinct signaling chains to transmit cytotoxic and proliferative signals, thereby achieving a synergistic effect. Notably, the activation of CAR-T cells depends on the simultaneous expression of both antigens by target tumor cells, effectively minimizing the risk of harming normal tissues [155]. This approach provides new insights for developing CAR-T therapies aimed at targeting malignant tumors of the CNS in the future.

In summary, we systematically reviewed the progress of preclinical and clinical studies of CAR-T cells in the treatment of gliomas, and CAR-T cell therapies for different targets showed their respective therapeutic potentials and challenges in targeting gliomas. These findings have helped us to gain a clearer understanding of the effectiveness and limitations of different targets in the treatment of gliomas. To further explore the practical clinical application of CAR-T cell therapy in glioma treatment, we summarised the data from the current preclinical studies and clinical studies, which are detailed in Tables 3 and 4. These data provide an important basis for evaluating the clinical efficacy of CAR-T cell therapy for gliomas and provide valuable guidance for future research directions and clinical practice.

**Table 3:** Preclinical study outcomes of CAR-T cell therapy for gliomas and medulloblastoma

Target	Disease type	Experimental Approach	Time	The structure of CAR-T cells
EGFRvIII	GBM	in vitro and in vivo	2021 [87]	EGFRvIII scFv-CD8stk-mCD28-CD3ζ
EGFRvIII	GBM	in vitro and in vivo	2023 [88]	EGFRvIII scFv-CD8α(H/M)-CD28-CD3ζ-Myc tag
EGFRvIII	GBM	in vitro and in vivo	2015 [89]	3C10scFv-CD8α hinge-4-1BB-CD3ζ
IL13Rα2	GBM	in vitro and in vivo	2024 [100]	CD8α SP-scFv IL-13Rα2-CD8α hinge-CD28 TM-CD28cyto-4-1BB-CD3ζ
HER2	GBM	in vitro	2019 [65]	HER2 scFv-hinge-CD28 and CD137-CD3ζ
GD2	GBM	in vitro and in vivo	2022 [111]	NA
GD2	GBM	in vitro and in vivo	2020 [115]	GD2 scFv-hinge-CD28 -CD3ζ
GD2	glioma	in vivo	2018 [117]	GD2 scFv-CD8 TM-4-1BB -CD3ζ
B7H3	DIPG	in vitro and in vivo	2024 [130]	B7H3 scFv-CD8α(H/M)-TMIGD2 -CD3ζ-P2A-hEGFRt
B7H3	GBM	in vitro and in vivo	2023 [129]	B7H3 scFv-CD8α hinge-4-1BB -CD3ζ-mCherry
EphA2	GBM	in vitro and in vivo	2018 [135]	EphA2 scFv-CD28 TM-CD28 -CD3ζ; EphA2 scFv-CD8 TM-4-1BB -CD3ζ; EphA2 scFv-CD28 TM-CD28 and 4-1BB -CD3ζ
EphA2	GBM	in vitro and in vivo	2021 [136]	EphA2 scFv-a-CD28 TM-CD28 and 4-1BB -CD3ζ; EphA2 scFv-b-CD28 TM-CD28 and 4-1BB -CD3ζ
P32	GBM	in vitro and in vivo	2021 [57]	P32 scFv-CD28-FcRγ -P2A-mCherry
CSPG4	GBM-NS	in vitro and in vivo	2018 [139]	CSPG4 scFv-CD8α(H/M)-CD28/4-1BB/CD28 and 4-1BB-CD3ζ
NKG2DL	glioma	in vitro and in vivo	2022 [143]	NA
NKG2DL	GBM	in vitro and in vivo	2019 [78]	NKG2D ECD-CD8 hinge and TM- 4-1BB -CD3ζ
CD70	GBM	in vitro and in vivo	2018 [79]	hCD27-4-1BB-CD3ζ-P2A-tT; mCD27-4-1BB-CD3ζ-P2A-tT
CD70	GBM	in vitro and in vivo	2021 [45]	CD70 scFv-CD8α-4-1BB -CD3ζ
CD70	GBM	in vitro and in vivo	2023 [80]	RVG29-CD70scFv-CD8 hinge-4-1BB -CD3ζ
CD133	GBM	in vitro and in vivo	2020 [147]	CD133scFv-Myc tag-CD8α-CD28 -CD3ζ
IL-13Rα2 TGF-β	GBM	in vitro and in vivo	2024 [149]	NA
EGFRvIII EphA2 IL13Rα2	GBM	in vitro and in vivo	2021 [154]	EGFRvIII scFv, EphA2 scFv and IL13 mutein scFv or IL13 mutein-G4Sx4-EphA2 scFv-CD8α hinge-4-1BB-CD3ζ
HER2	MB	in vitro and in vivo	2018 [156]	4D5 anti-HER2 scFv-αCD8 hinge TM-4-1BB -CD3ζ
GD2	MB	in vitro and in vivo	2024 [157]	FKBP12-F36V-iCasp9-scFv(14g2a)-CD28TM-CD28 and 4-1BB -CD3ζ
B7H3	MB	in vitro and in vivo	2019 [158]	CD276 scFv-CD8 TM-4-1BB -CD3ζ
B7H3	MB	in vitro and in vivo	2021 [159]	B7H3 scFv-CD28(H/TM)-CD28 -CD3ζ

*GBM *Glioblastoma, *MB *Medulloblastoma, *scFv *Single-chain variable fragment, *TM *Transmembrane

**Table 4:** Clinical study outcomes of CAR-T cell therapy for gliomas

Disease type	Target	Registration Number	Research Phase	Partici-pants	Time	Research Institution	Authors	Clinical outcomes	Adverse events
GBM	EGFRvIII	NCT02209376	phase 1	10	2017	Perelman School of Medicine at the University of Pennsylvania, USA	O’Rourke DM, et al [44]	Median OS: 8 months	CRS:0; ICANS:30%; off-tumor toxicity:0
GBM	EGFRvIII	NCT01454596	phase 1	18	2019	National Cancer Institute, National Institutes of Health	Goff SL, et al [90]	Median PFS: 1.3 months; Median OS: 6.9 months	severe hypoxia:2 patients; transient hematologic toxicities: 100%
GBM	IL13Rα2	NCT00730613	phase 1	3	2015	City of Hope Beckman Research Institute and Medical Center, USA	Brown CE, et al [101]	Evidence of a transient antitumor response was observed in two patients.	10^7^ or 5×10^7^ T cell dose: Grade 3 or higher adverse events:0; 10^8^ T cell dose: one Grade 3 headache, one Grade 3 neurologic- event
GBM	IL13Rα2	NCT02208362	phase 1	1	2016	City of Hope Beckman Research Institute and Medical Center, USA	Brown CE, et al [13]	PFS:7.5 months	toxic effects of grade 3 or higher: none
high-grade glioma	IL13Rα2	NCT02208362	phase 1	58	2024	City of Hope Beckman Research Institute and Medical Center, USA	Brown CE, et al [102]	Median OS: 8 months; the subset with rGBM:7.7 months; SD or better:50%; PR:2 patients; CR: 1 patient	Grade 3 and above toxicities:35%
GBM	IL13Rα2	NCT01082926	phase 1	6	2022	City of Hope Beckman Research Institute and Medical Center, USA	Brown CE, et al [103]	four showed signs of transient tumor reduction and/or necrosis	All ≤ grade 3
GBM	HER2	NCT01109095	phase 1	17	2017	Baylor College of Medicine, Houston Methodist Hospital, and Texas Children’s Hospital, USA	Ahmed N, et al [66]	Median OS: 11.1 months; PR and SD:8 patients	NA
CNS tumors	HER2	NCT03500991	phase 1	3	2021	Seattle Children’s Research Institute, USA	Vitanza NA, et al [110]	well tolerated	headache, pain or transient worsening of a baseline neurologic deficit
GBM	GD2	NCT03170141	phase 1	8	2023	Shenzhen Hospital, Southern Medical University, China	Liu Z, et al [118]	Median OS: 10 months; 3–24 months:50%PR; 6–23 months:37.5% PD; 4 months:12.5% SD	No severe adverse effects were observed
DIPG or spinal cord diff-use DMG	GD2	NCT04196413	phase 1	4	2022	Stanford Center for Cancer Cell Therapy, Stanford Cancer Institute, Stanford University, USA	Majzner RG, et al [119]	Clinical and radiographic improvement:75%	TIAN
H3K27M-mutated DMG; MD; AT/RT	GD2	NCT04099797	phase 1	11	2024	Baylor College of Medicine, USA	Lin FY, et al [121]	C7R-GD2 CAR-T: a median duration of neurofunctional improvement:5 months, PR and SD:88%; GD2 CAR-T: duration of neurofunctional improvement for less than 3 weeks	C7R-GD2 CAR-T: 1 TIAN:88%; CRS:75%; GD2 CAR-T: no CRS and TIAN
DIPG	B7H3	NCT04185038	phase 1	5	2023	Seattle Children’s Research Institute, USA	Vitanza NA, et al [131]	one patient exhibited continuous clinical and radiological improvement over a 12-month follow-up period	headache (3/3), nausea/vomiting (3/3), and fever (3/3),gait disturbance, dysphagia
GBM	EphA2	NCT03423992	phase 1	3	2021	Xuanwu Hospital, Capital Medical University, China	Lin Q, et al [137]	SD:1 patient; PD:2 patients; OS:86-181d	2 CRS accompanied by pulmonary edema: 2 patients
GBM	EGFRvIII and IL13Rα2	NCT05168423	phase 1	6	2024	University of Pennsylvania Perelman School of Medicine, USA.	Bagley SJ, et al [150]	reductions in tumor size:100%; none met criteria for ORR	Dose-limiting toxicity: 1 patient

*GBM *Glioblastoma, *OS *Overall survival, *CRS *Cytokine release syndrome, *ICANS *Immune effector cell-associated neurotoxicity syndrome, *PFS *Progression-free survival, *rGBM *Recurrent glioblastoma, *SD *Stable disease, *PR *Partial response, *CR *Complete response, *PD *Progressive disease, *TIAN *Tumor-associated inflammatory adverse events, *ORR *Overall response rate

### Medulloblastoma

Medulloblastoma(MB) is one of the most prevalent malignant brain tumors in children and is classified into four distinct subtypes based on molecular characteristics: WNT, SHH, G3, and G4 [160]. WNT MBs are typically associated with mutations in the CTNNB1 gene, nuclear immunohistochemical staining positive for β-catenin, and monosomy six (deletion of one copy of chromosome 6 in the tumor). These changes are associated with the aberrant activation of the WNT signaling pathway, which is characterized by this subtype having the most favorable prognosis among all MBs. SHH MBs are strongly associated with the abnormal activation of the Sonic Hedgehog signaling pathway. Studies have demonstrated that individuals with mutations in the PTCH, SMO, or SUFU genes are predisposed to this subtype, with the most common histological subtypes being the classic and desmoplastic variants. In comparison, the tumorigenic molecular mechanisms of the G3 group of MBs remain unclear, characterized by MYC amplification, which is rarely observed in adults and primarily occurs in infants and children, representing the worst prognosis among all subtypes. The G4 group of MBs is the most prevalent molecular subtype, accounting for approximately 35% of all MBs, with characteristic mutations frequently affecting the KDM6A gene [161, 162]. Standard treatment options include surgical intervention, radiotherapy, and chemotherapy; however, these approaches frequently result in considerable neurological and endocrine damage [163]. Research indicates that MB exhibits high expression levels of EphA2, HER2, and IL-13Rα2 [164]. These antigens represent promising targets for CAR-T cell immunotherapy, offering new avenues for the treatment of MB.

#### HER2

Studies have demonstrated that both MB and posterior fossa A(PFA) ependymoma specifically overexpress EphA2, HER2, and IL-13Rα2 [164]. HER2 CAR-T cells exhibited significant anti-tumor activity in treating MB and effectively cleared xenografted tumors in a mouse model via both regional and intravenous administration, with the dose required for regional administration being significantly lower than that for intravenous. Furthermore, non-human primate studies confirmed that ventricular administration of HER2 CAR-T cells was feasible and safe, with no systemic toxicity observed [156]. These findings provide strong support for future clinical trials involving direct injection of HER2 CAR-T cells into the CSF for patients with MB. Treatment with EphA2-targeted CAR-T cells significantly extended the survival of tumor-bearing mice and effectively inhibited MB metastasis to the spinal cord. Repeated local administration via intraventricular injection resulted in improved therapeutic outcomes, with EphA2 CAR-T monotherapy showing superior efficacy compared to the EphA2/HER2/IL-13Rα2 trivalent CAR-T therapy. Additionally, the study concluded that the combination of Azacytidine with CAR-T cells further enhances their efficacy in tumor clearance in mice [164]. However, as this study did not comprehensively evaluate systemic toxicity, additional research is warranted to thoroughly investigate its long-term safety and potential adverse effects.

#### GD2

GD2 is a potential antigen for MB and is expressed in approximately 80% of MB patient samples, despite its heterogeneous expression [157]. In vitro co-culture assays demonstrated that GD2 CAR-T cells exhibited significant anti-tumour activity. In the NSG mouse model of MB, intravenous injection of GD2 CAR-T cells significantly inhibited tumor growth and prolonged the OS of mice. To mitigate the potential toxicities associated with GD2 CAR-T cells, the researchers introduced a suicide gene, inducible caspase 9 (iC9), into the CAR-T cells as a safety switch [165]. The gene can be activated by the chemical dimeriser AP1903, which rapidly eliminates CAR-T cells from circulation and the brain, thereby reducing the risk of toxicity [157]. The results of this study provide robust evidence supporting the application of GD2 CAR-T cells in clinical trials.

#### B7H3

B7H3 is highly expressed in pediatric CNS tumor tissues. CAR-T cells targeting B7H3 were significantly activated when co-cultured with MB cell lines in vitro, as evidenced by the secretion of cytokines including TNF-α, IL-2, and IFN-γ. Furthermore, studies demonstrated that tail vein-injected B7H3 CAR-T cells crossed the BBB and successfully infiltrated the brain of DAOY MB and c-MYC-amplified group 3 MB xenograft mouse models, resulting in tumor clearance and significantly prolonged survival [158]. Local administration and systemic infusion of B7H3 CAR-T cells in a patient derived orthotopic xenograft (PDOX) MB mouse model resulted in significantly increased mouse survival rates [159]. These findings indicate that B7H3 CAR-T cells exhibit substantial anti-tumor efficacy and demonstrate effective BBB penetration, offering robust evidence for their potential application in treating pediatric MB. Nonetheless, additional clinical trials are required to confirm their long-term efficacy and safety.

### Tumours of the lymphohematopoietic system involving the CNS

#### Non-hodgkin lymphoma

Central nervous system lymphoma (CNSL), comprising primary CNSL (PCNSL) and secondary CNSL (SCNSL), has a worse prognosis than extracerebral lymphoma, with a 5-year survival rate of only 29.9%. The overall prognosis remains poor, despite advances in high-dose cytarabine and methotrexate treatments, which have significantly improved survival [166]. Moreover, patients with CNSL are frequently excluded from key clinical trials owing to concerns about severe adverse events, such as ICANS, that may arise following CAR-T therapy [167]. These challenges highlight the particularly complex nature of CNSL treatment.

In recent years, several studies have demonstrated the feasibility and safety of CAR-T cell therapy in patients with CNS lymphoma [168–174]. A single-center retrospective analysis demonstrated that 85.7% of seven patients with SCNSL who underwent CAR-T cell therapy achieved CR by day 28, whereas bridging with whole-brain radiotherapy (WBRT) did not significantly elevate the risk of ICANS, indicating that CAR-T cell therapy remains a viable option for patients with SCNSL [175]. Alcantara M et al. reported that a study involving nine patients with PCNSL treated with tisa-cel or axi-cel demonstrated that the treatment regimen was well tolerated. The CR rate was 55.6% at three months of treatment, and the median PFS was 122 days [176]. The efficacy of tisagenlecleucel in patients with highly refractory PCNSL was evaluated in a phase 1/2 clinical trial, demonstrating a response rate of 58.3%, with 50% of patients achieving CR, along with a manageable safety profile [177]. The study indicated that tisagenlecleucel was well tolerated and effective in this highly refractory PCNSL cohort. A clinical trial of 21 patients with CD19 + PCNSL/SCNSL reported an overall response rate (ORR) of 67%, with CR in 29% and PR in 38% of patients at day 28 post-infusion. The median PFS was three months, whereas the median OS for patients with PCNSL reached 15 months, significantly outperforming that of patients with SCNSL. Regarding adverse events, the incidence of CRS and ICANS was 76% and 33%, respectively. Additionally, the study found that CAR-T cells successfully crossed the BBB and were detectable in the cerebrospinal fluid. Enhanced cytotoxic activity in the CNS was strongly correlated with a higher proportion of CD8 + T cells, a lower proportion of Tregs, and elevated expression levels of IL-7 [178]. One of the largest global cohort studies on CAR-T cell therapy for PCNSL demonstrated substantial efficacy in recurrent PCNSL, with 64% of patients achieving CR. Among those who attained either CR or PR following CAR-T cell infusion, the 1-year relapse-free survival (RFS) rate was 79% [179]. Data from six US centers indicated that CD19 CAR-T therapy for patients with CNS involvement in diffuse large B-cell lymphoma (DLBCL) achieved a 92% CR rate at three months, with only 8% of patients experiencing PD. The incidence of CRS was 91.67%, with all cases classified as grade 1–2, whereas the incidence of ICANS was 83.33%, with 58% of cases classified as grade 3–4. The study demonstrated a high CR rate and generally manageable adverse events; however, the relapse rate remained notably high [180]. Alsouqi et al. reported the efficacy of commercial CAR-T cell therapy in 113 patients with SCNSL, encompassing individuals with either active SCNSL or a history of SCNSL. Among the 80 patients with an assessable CNS response, the ORR at one month was 68%, and the CR rate was 34%. The median PFS in the subgroup of patients with active SCNSL was 2.9 months, and the median OS was 8.6 months. CRS occurred in 75% of patients, predominantly of grades 1–2, whereas ICANS was observed in 56% of the cohort [181]. These adverse reactions underscore the necessity for a thorough evaluation of patient tolerability and safety in the context of CAR-T cell therapy for SCNSL. A multicenter retrospective analysis of relma-cel in Chinese patients with relapsed/refractory CNSL(R/R CNSL) demonstrated significant efficacy, with a 1-year PFS rate of 64.4% and an ORR of 90.9%, suggesting the role of BTK inhibitors or PD-1 inhibitors in facilitating CAR-T cell re-expansion. The study emphasized the importance of early application of CAR-T cell therapy as consolidation therapy in patients sensitive to salvage therapy [182]. These results suggest that CAR-T cell therapy demonstrates significant potential in CNSL treatment, which is crucial for patients who do not respond well to conventional therapy. However, further optimization of treatment regimens and improved safety management are required to increase long-term efficacy and reduce the incidence of adverse events.

CD19 or CD20 CAR-T cell therapy for CNS lymphoma has demonstrated promising efficacy. A study administering this therapy to seven patients with CNS lymphoma reported CR in four patients and PR in three patients [183]. A study retrospectively analyzed 15 patients with R/R CNSL who were treated with various CAR-T cell therapies, including single CD19 CAR-T, CD19 sequential CD20 CAR-T, and CD19/CD22 dual-target CAR-T. This study found an ORR of 73.3% (11/15), with 60% of patients achieving CR and 13.3% achieving PR. Regarding safety, 73.3% of patients experienced grade 1–2 CRS, whereas 20% developed ICANS, including one case of grade 4 ICANS. These CAR-T cell therapies demonstrated promising anti-tumor efficacy and acceptable side effects in SCNSL, highlighting their potential for treating this condition. Wu J et al. reported the results of a study evaluating sequential CD19/22 CAR-T therapy following ASCT in patients with CNSL. The study demonstrated an ORR of 81.81% and a CR rate of 54.55%, with manageable adverse effects. Notably, no grade 3–4 CRS occurred, and only one patient experienced severe ICANS, underscoring the promising long-term efficacy of the treatment [184]. Future studies should explore the effectiveness of CAR-T cell therapy for CD19 and other targets in CNSL and evaluate its safety and tolerability in practical clinical applications. This may aid in providing more effective treatment options for patients with CNSL.

#### Acute lymphoblastic leukemia

Despite the remarkable results of CD19 CAR-T cells in the treatment of acute B-cell lymphoblastic leukemia [185–187], few studies have investigated CAR-T cell therapy for patients with B-ALL with CNSL, primarily owing to concerns regarding poor treatment response and associated neurotoxicity risk.

A study evaluating the efficacy and safety of CD19-targeted CAR-T cell therapy in 48 patients with R/R B-ALL with CNS involvement indicated a remission rate of 85.4% in the CNS, with a median event-free survival (EFS) of 8.7 months and a median OS of 16.0 months during a median follow-up of 11.5 months. The recurrence rate of CNS involvement was 11.3%. The therapy was generally well tolerated, with an 18.8% incidence of CRS and a 22.9% incidence of grade 3–4 ICANS, both with manageable toxicity [188]. These findings suggest that CD19-targeted CAR-T cell therapy exhibits a favorable response rate in CNS involvement and may be an effective and manageable treatment option for patients with CNSL who were previously deemed ineligible for the therapy. Tan Y et al. retrospectively analyzed the outcomes of 12 pediatric patients with low (< 20/µL blasts in the CSF) or high (blasts in CSF or significant intracranial mass) disease-burden CNS B-ALL treated with CD19 CAR-T cells. The study reported that 91.7% of patients achieved CR within 30 days of treatment, with a 6-month leukemia-free survival (LFS) rate of 81.8%. However, four of the high-burden patients developed severe ICANS, manifesting as persistent cerebral edema and seizures, necessitating intensive intervention [189]. The study suggests that CAR-T cells are effective in eliminating low- and high-burden CNS B-ALL but may result in severe neurotoxicity that necessitates active management in high-burden cases.

Despite some challenges, CAR-T cell therapy has shown considerable therapeutic potential in managing lymphohematopoietic tumors affecting the CNS. We compiled data from prominent ongoing clinical trials to better understand the efficacy of this therapeutic approach. Table 5 presents the results of clinical trials of CAR-T cell therapy across various tumor types, offering a crucial reference for future research and clinical practice.

**Table 5:** Clinical study outcomes of CAR-T cell therapy for tumors of the lymphohematopoietic system involving the CNS

Disease type	Target	Product Types	Center	Partici-pants	Time	Authors	Research Institution	Clinical outcomes	Adverse events
SCNSL	CD19	tisa-cel	singlecenter	8	2019	Frigault MJ, et al [173]	Massachusetts General Hospital Cancer Center, USA	1 month: CR:25%,PR:25%,PD:50%	>1 ICANS:0
SCNSL	CD19	axi-cel, tisa-cel	singlecenter	7	2021	Ahmed G, et al [175]	Medical College of Wisconsin, USA	At day 28: CR:85.7%,PD:14.3%; The median PFS:83 days(28–219); The median OS:129 days(32–219)	no ICANS:3 patients; 1 or 3 ICANS:2 patients
PCNSL	CD19	investigational product	singlecenter	5	2021	Siddiqi T, et al [169]	City of Hope Medical Center, USA	CR:60%,SD:40%	1 CRS:100%; 1 ICANS:100%
PCNSL	CD19	axi-cel, tisa-cel	multicenter	9	2022	Alcantara M, et al [176]	PSL Research University, France	1 month: ORR:66.7%,CR:33.3%; 3 months: ORR:66.7%;CR:55.6%; 6 months: OS:89%,PFS:44%,DoR:67%; The median PFS:122 days	CRS:7 patients; ICANS:5 patients
PCNSL	CD19	tisa-cel	singlecenter	12	2022	Frigault MJ, et al [177]	Massachusetts General Hospital and Harvard Medical School, USA	ORR:58.3%,CR:50%	CRS:58.3%; ICANS:41.6%
SCNSL	CD19	investigational product	singlecenter	10	2022	Karschnia P, et al [174]	University Hospital, LMU, Germany	1 month disease response:70%; The median PFS:7 months	ICANS:60%
PCNSL/SCNSL	CD19	axi-cel, tisa-cel	singlecenter	21	2023	Lacan C, et al [178]	PitiéSalpêtrière Hospital, France	Persistent response:38%(6 CR 2 PR); The median OS in PCNSL:15 months; The median PFS:3 months	CRS:16(1 grade 3); ICANS:7(2 grade ≥ 3)
SCNSL	CD19	investigational product	multicenter	10	2023	Ryan CE, et al [170]	Dana-Farber Cancer Institute, USA	ORR in the CNS:86%; 1 year PFS:47%	CRS:90%; ICANS:70%
PCNSL/SCNSL	CD19	tisa-cel, axi-cel, liso-cel	singlecenter	44	2023	Karschnia P, et al [190]	Harvard Medical School, USA	ORR:68.9%;CR:40.0%	ICANS: In PCNSL:44.4%; In SCNSL:66.7%
SCNSL	CD19	axi-cel, tisa-cel	multicenter	28	2023	Ayuk F, et al [171]	University Medical Center Hamburg-Eppendorf, Germany	CR:32%;PR:32%;SD:11%;PD:25%The median OS:21 months; The median PFS:3.8 months	CRS:86%; ICANS:46%
PCNSL	CD19	axi-cel, tisa-cel	multicenter	25	2024	Choquet S, et al [179]	Hôpital Pitié-Salpêtrière, France	The best response:64%; 1 year relapse-free survival:79%; The median OS:21.2 months;	CRS:92%; ICANS:68%
MCL with SCNS involvement	CD19	Brexu-cel, investigational product	multicenter	12	2024	Ahmed G, et al [180]	Medical College of Wisconsin, USA	CNS response: 1 month: CR:92%;PR:8%; 3 months: CR:92%:PD:8%; 6 months PFS:50%;OS:75%; 12 monthsPFS:25%;OS:63%; incidence of relapse:50%	1–2 CRS:91.67%; ICANS:83.33%(3–4 ICANS:58%)
SCNSL	CD19	tisa-cel, axi-cel, liso-cel	multicenter	113	2024	Alsouqi A, et al [181]	University of Pittsburgh Medical Center, USA	1 month ORR:68%,CR:34%; subgroup of patients with active SCNSL: The median PFS :2.9 months; The median OS:8.6 months	CRS:75%; ICANS:56%
PCNSL/SCNSL	CD19	relma-cel	multicenter	22	2024	Yu W, et al [182]	Ruijin Hospital Affiliated to Shanghai Jiao Tong University School of Medicine, China	the best ORR:90.9%; BCR rate:68.2%; the estimated 1-year PFS:64.4%;DOR:71.5%;OS:79.2%	CRS:72.9%; ICANS:36.4%
PCNSL/SCNSL	CD19/CD20	investigational product	singlecenter	7	2022	Shumilov E, et al [183]	The Third Xiangya Hospital of Central South University, China	CR:4 patients; PR:3 patients; The median duration of CR:22.4 months	1 CRS:71.43%, 2 CRS:28.57%; None ICANS; Hematological toxicities:71.43%
SCNSL	CD19 CD20 CD22	investigational product	multicenter	15	2022	Zhang H, et al [191]	The Affiliated Hospital of Xuzhou Medical University, China	ORR:73.3%;CR:60%;PR:13.3%; The median OS:9 months; The median PFS:4 months	1–2 CRS:73.3%; ICANS:20%
PCNSL/SCNSL	CD19 CD22	investigational product	singlecenter	13	2021	Wu J, et al [184]	Tongji Hospital, Tongji Medical College, Huazhong University of Science and Technology, China	ORR:81.81%; CR:54.55%; 1 year PFS:74.59%; 1 year OS: 82.50%	3–4 CRS:0; ICANS:1 patients
B-ALL	CD19	investigational product	singlecenter	12	2021	Tan Y, et al [189]	Beijing Boren Hospital, China	a month: CR:91.7%; Sustained remission:75%; 6-months LFS:81.8%;	3–4 ICANS:33.3%
B-ALL	CD19	investigational product	multicenter	48	2022	Qi Y, et al [188]	Xuzhou Medical University, China	ORR:85.8%; The median EFS:8.7 months; The median OS:16 months	3 CRS:18.8%; 3–4 ICANS:22.9%

*SCNSL *Secondary central nervous system lymphoma, *PCNSL *Primary central nervous system lymphoma, *CR *Complete response, *PR *Partial response, *PD *Progressive disease, *CRS *Cytokine release syndrome, *ICANS *Immune effector cell-associated neurotoxicity syndrome, *PFS *Progression-free survival, *OS *Overall survival, *SD *Stable disease, *ORR *Overall response rate, *DoR *Duration of response

**Fig. 3:**
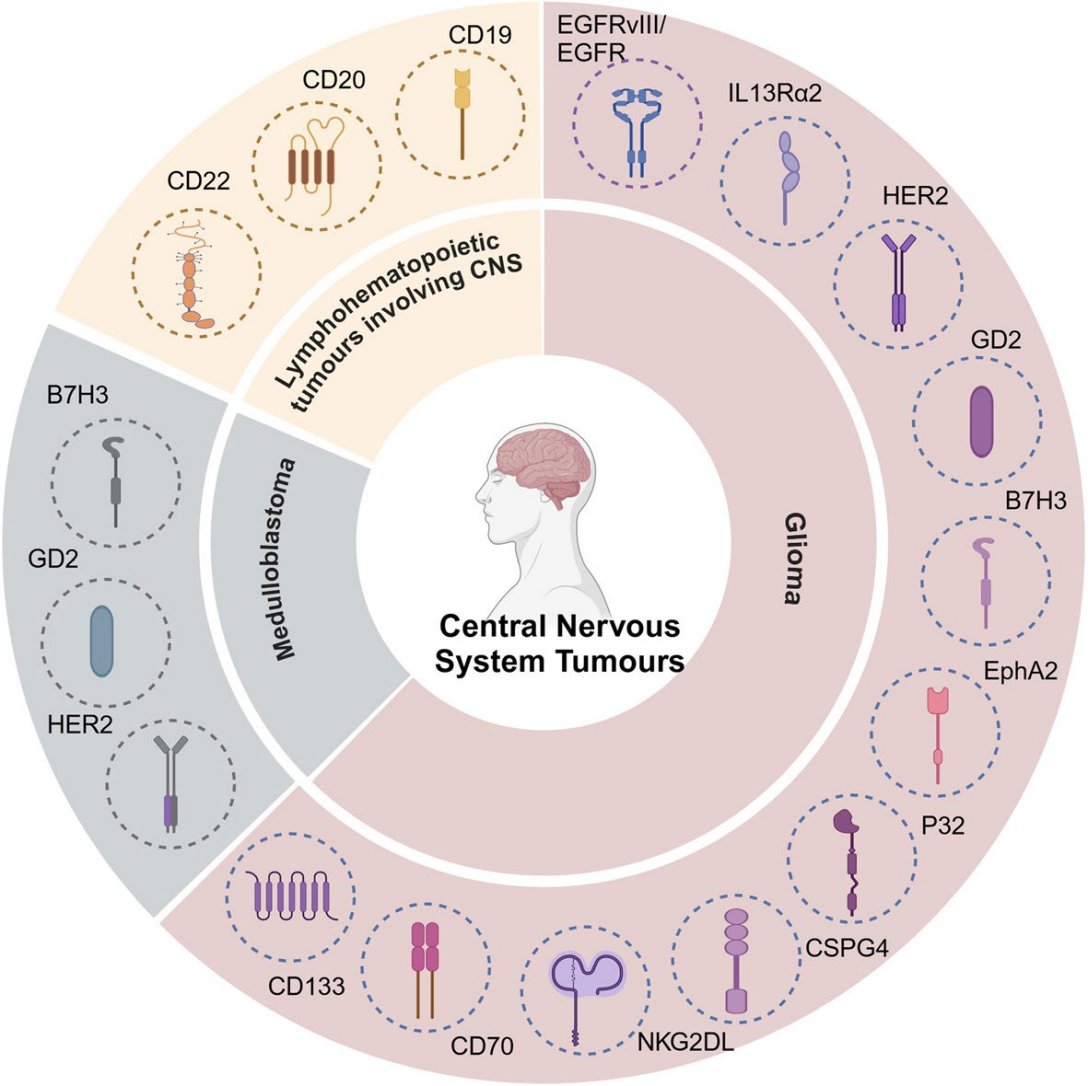
Illustrates the common targets of CAR-T cells in CNS tumors. In glioma, these targets include EGFRvIII/EGFR, IL-13Rα2, HER2, GD2, B7H3, EphA2, P32, CSPG4, NKG2DL, CD70, and CD133. In medulloblastoma, the principal targets are HER2, GD2, and B7H3. In lymphohematopoietic tumors involving the CNS, common targets include CD19, CD20, and CD22

CAR-T cells targeting various antigens (Fig. 3) have demonstrated promising feasibility and safety profiles in CNS tumors. Certain targets such as P32, CSPG4, NKG2DL, CD70, and CD133 remain under investigation in preclinical studies, whereas others including EGFRvIII/EGFR, IL-13Rα2, HER2, GD2, B7H3, EphA2, and CD19 have been validated in clinical trials. Notably, CAR-T cell therapies targeting CD19 have been commercialized and are currently being utilized to treat hematological malignancies, including those involving the CNS. These findings suggest that CAR-T cell therapy holds significant promise in the treatment of CNS tumors, warranting further in-depth research and exploration.

Moreover, although CAR-T cell therapy has demonstrated potential efficacy in treating CNS tumors, several clinical trials have been discontinued for various reasons, including safety concerns (such as severe or potentially fatal neurotoxicity), insufficient efficacy, challenges in patient recruitment, and funding disruptions (Table 6). In this context, studies focused on hematologic malignancies offer valuable insights. For instance, patients treated with CD30 CAR-T therapy for CD30 + lymphoma experienced rashes and prolonged toxicities, ultimately resulting in the premature termination of the trial [192]. This phenomenon raises concerns regarding the safety of subsequent research, particularly in the treatment of CNS tumors, where neurotoxicity may be misinterpreted as the patient’s underlying neurological symptoms, thereby complicating assessment and management. Consequently, future research should prioritize optimizing CAR-T cell design, enhancing the specificity of target selection, and minimizing adverse reactions to promote the safe and effective application of this therapy in CNS tumors.

**Table 6:** Terminated CAR-T cell therapy clinical trials and corresponding reasons for termination (https://clinicaltrials.gov/)

NCT	Study Title	Phase	Disease type	Target	Research Institutions	Termination reasons
NCT02664363	EGFRvIII CAR T Cells for Newly-Diagnosed WHO Grade IV Malignant Glioma	phase 1	glioma	EGFRvIII	The Preston Robert Tisch Brain Tumor Center at Duke	Study funding ended
NCT03283631	Intracerebral EGFR-vIII CAR-T Cells for Recurrent GBM	phase 1	GBM	EGFRvIII	Duke University Medical Center	The trial was suspended pending the review and approval of an amendment to reduce the anticipated number of participants.
NCT02209376	Autologous T Cells Redirected to EGFRVIII-With a Chimeric Antigen Receptor in Patients With EGFRVIII + Glioblastoma	phase 1	GBM	EGFRvIII	University of Pennsylvania	Sponsor decision to terminate prior to completion to pursue combination therapies.
NCT03252171	CAR-T Cell Immunotherapy for GD2 Positive Glioma Patients	phase 1/2	glioma	GD2	Fuda Cancer Hospital, Guangzhou	Project terminated due to revision of local regulations
NCT03049449.	T Cells Expressing a Fully-Human Anti-CD30 Chimeric Antigen Receptor for Treating CD30-Expressing Lymphomas	phase 1	lymphomas	CD30	National Institutes of Health Clinical Center	The trial was halted early because of toxicity
NCT02535364	Study Evaluating the Efficacy and Safety of JCAR015 in Adult B-cell Acute Lymphoblastic Leukemia (B-ALL)	phase 2	B-ALL	CD19	University of Alabama Birmingham Comprehensive Cancer Center	Safety reasons
NCT02706405	JCAR014 and Durvalumab in Treating Patients With Relapsed or Refractory B-cell Non-Hodgkin Lymphoma	NA	NHL	CD19	Fred Hutchinson Cancer Center	Terminated due to slow accrual
NCT04844086	RPM CD19-mbIL15-CAR-T Cells in Patient With Advanced Lymphoid Malignancies	phase 1	Lymphoid malignancies	CD19	National Taiwan University Hospital	CAR-T manufacturing technology can not meet the dose requirement for clinical patients
NCT04789408	Study of KITE-222 in Participants With Relapsed/​Refractory Acute Myeloid Leukemia	phase 1	AML	CD19	Stanford Cancer Center	Study was terminated due to futility
NCT04219319	LCAR-T2C CAR-T Cells in Relapsed or Refractory CD4+ T-cell Lymphoma	phase 1	T-cell lymphoma	CD4	The First Affiliated Hospital with Nanjing Medical University	Both the sponsors and collaborator are considering terminating the study
NCT04142619	Study Evaluating Safety and Efficacy of UCART Targeting CS1 in Patients With Relapsed/​Refractory Multiple Myeloma (MELANI-01)	phase 1	MM	CS1	UCSF Medical Center- Helen Diller Family Comprehensive Cancer Center	The trial was discontinued due to sponsor’s decision and not a consequence of any safety concern
NCT03743246	A Study to Evaluate the Safety and Efficacy of JCAR017 in Pediatric Subjects With Relapsed/​Refractory (r/​r) B-cell Acute Lymphoblastic Leukemia (B-ALL) and B-cell Non-Hodgkin Lymphoma (B-NHL)	phase 1/2	B-ALL; B-NHL	CD19	Local Institution, USA	Absence of significant therapeutic benefit over existing therapies
NCT03672851	Study Evaluating Safety and Efficacy of CAR-T Cells Targeting CD123 in Patients With Acute Leukemia	phase 1	AL	CD123	Second Affiliated Hospital of Xi’an Jiaotong University	Adverse effect
NCT03593109	A Single-center Clinical Study to Evaluate the Safety, Tolerability, and Efficacy of LCAR-L10D Cell Formulations Targeting CD19 and CD22 in Patients With CD19- and/​or CD22-Positive Relapsed/​Refractory B-cell Lymphoma	phase 1	B-cell Lymphoma	CD19;CD22	Second Affiliated Hospital of Xi’an Jiaotong University	Suitable patients were not recruited
NCT03473457	CAR-T Cells Therapy in Relapsed/​Refractory Acute Myeloid Leukemia (AML)	NA	AML	CD33;CD38;CD56;CD123;CD117;CD133;CD34	Southern Medical University Zhujiang Hospital	The therapeutic effect was not as expected
NCT03473496	CAR-T Cells Therapy in Relapsed/​Refractory Multiple Myeloma (MM)	NA	MM	BCMA; CD138;CD56;CD38	Southern Medical University Zhujiang Hospital	After CAR-T treatment, the benefit is not significant, and it is difficult to enroll
NCT03318861	Study to Evaluate the Safety and Efficacy of KITE-585 in Participants With Relapsed/​Refractory Multiple Myeloma	phase 1	MM	BCMA	David Geffen School of Medicine at UCLA	The study was terminated due to lack of efficacy
NCT03287804	APRIL CAR T Cells (AUTO2) Targeting BCMA and TACI for the Treatment of Multiple Myeloma (APRIL)	phase 1/2	MM	BCMA; TACI	VU University Medical Centre Amsterdam	Preliminary efficacy seen to date following treatment with AUTO2 has been determined not sufficient to warrant further development
NCT02588456	Pilot Study of Autologous Anti-CD22 Chimeric Antigen Receptor Redirected T Cells In Patients With Chemotherapy Resistant Or Refractory Acute Lymphoblastic Leukemia	phase 1	ALL	CD22	Abramson Cancer Center of the University of Pennsylvania	This study was terminated due to lack of efficacy

*GBM *Glioblastoma, *B-ALL *B-cell acute lymphoblastic leukemia, *NHL *Non-hodgkin lymphoma, *AML *Acute myeloid leukemia, *MM *Multiple myeloma, *AL *Acute leukemia

## Strategies to enhance the efficacy of CAR-T cell therapy in the CNS

When discussing strategies to enhance the efficacy of CAR-T cell therapy for CNS tumors, it is essential to address both the unique challenges of this approach and draw lessons from the experience with monoclonal antibodies (mAbs) in cancer treatment. Despite significant mechanistic differences between mAbs and CAR-T cells—such as the short half-life and repeated administration required for mAbs compared to CAR-T cells, which as a “living drug” can proliferate and persist after infusion—there are shared principles [193]. First, the success of mAbs underscores the critical importance of selecting the appropriate antigen target [194], a principle equally applicable to CAR-T cell therapy. Second, the development of mAbs has revealed that tumor cells can develop resistance through mechanisms like antigen escape or alterations in the tumor microenvironment [195, 196], which are crucial considerations for the design and optimization of CAR-T cells. Expanding upon the use of mAbs, antibody-drug conjugates (ADCs) have significantly enhanced anti-tumor efficacy by combining the specificity of mAbs with potent cytotoxic drugs [197]. Although CAR-T cells cannot directly carry cytotoxic drugs, they can be combined with other therapies, such as PD-1 inhibitors or small-molecule targeted agents, or engineered to secrete cytokines, improving efficacy and reducing resistance. Furthermore, multispecific antibodies, which target two or more tumor-associated antigens, have demonstrated enhanced anti-cancer effects [198]. This concept suggests that multi-target CAR-T cells could reduce tumor escape and improve therapeutic outcomes. Similar to mAbs, the clinical application of CAR-T therapy requires robust clinical data. For instance, while bevacizumab, a VEGF monoclonal antibody, has been shown to prolong PFS in GBM, its lack of OS benefit remains controversial [199], and it is not approved in Europe for this indication [200]. This highlights the necessity for rigorous clinical validation of CAR-T therapies, which must advance beyond preclinical stages. Therefore, drawing on the experience of mAbs in cancer treatment can help to advance the clinical application of CAR-T cell therapies in CNS tumors and improve therapeutic outcomes.

### Combination with checkpoint blockade therapy

CAR-T cell exhaustion is a key factor affecting treatment efficacy. CNS tumors typically exhibit high expression of immunosuppressive molecules such as programmed death ligand 1 (PD-L1) on their surface, and the binding of these molecules to programmed death 1 (PD-1) on CAR-T cells results in CAR-T cell exhaustion. Blocking these inhibitory molecules can synergistically enhance anti-tumor efficacy and significantly boost the killing capacity of CAR-T cells [201]. One study subjected T cells to multi-gene editing using the CRISPR-Cas9 system, successfully knocking out the endogenous T cell receptor (TRAC), β2 microglobulin (B2M), and PD-1 genes, thereby generating general-purpose EGFRvIII CAR-T cells that were tolerant to PD-1 inhibition. The results demonstrated that PD-1 knockout significantly enhanced CAR-T cell activity and tumor-killing capacity in a preclinical model of human GBM [202]. However, relevant studies remain limited, and their definitive clinical effects require further validation.

### Secretion of cytokines

Cytokines demonstrate significant potential in enhancing CAR-T cell therapy for tumors. Reserach indicated that the transgenic expression of IL-15 can enhance CAR-T cell proliferation in vitro and prolong their persistence in vivo, thereby enhancing their antitumor activity. For instance, in a preclinical study targeting IL13Rα2-positive gliomas, the expression of IL-15 significantly extended the survival of IL13Rα2-CAR T cells and improved their efficacy against gliomas. However, the research also revealed that, despite the improved persistence of CAR-T cells, tumors post-treatment exhibited antigen loss. This suggests that while employing IL-15 to enhance CAR-T cell therapy, it is essential to adopt multi-target strategies to address the challenges posed by antigen loss [203]. Currently, clinical trials combining IL-15 with CAR-T therapy have been initiated in hematological malignancies [204]; however, in the context of glioma treatment, it remains at the preclinical stage, and its clinical efficacy has yet to be validated. Additionally, CAR-T cells engineered to secrete IL-12 effectively countered regulatory T cell-mediated immunosuppression and eradicated systemic tumors, without the need for pretreatment. For patients who cannot tolerate traditional lymphodepleting preconditioning, IL-12-CAR-T cells represent a promising alternative strategy [205]. However, despite exhibiting substantial theoretical antitumor activity, multiple clinical studies targeting advanced tumors have reported limited efficacy and significant treatment-related toxicities, indicating that the clinical application of IL-12 continues to face challenges [206]. In contrast, IL-18, a pro-inflammatory cytokine, plays a crucial role in enhancing CAR-T cell efficacy. It promotes CAR-T cell proliferation, facilitates CD4 + T cell assistance for CD8 + T cells, and increases the levels of IFN-γ secretion. In xenograft models, CAR-T cells secreting IL-18 demonstrate potent tumor-killing capabilities, further confirming their critical role in enhancing the efficacy of CAR-T cell therapy [207, 208]. Compared to IL-12, IL-18, a monomeric cytokine, presents a relatively lower risk of toxicity, providing certain advantages in clinical applications [207]. Moreover, CAR-T cells secreting IL-7 and CCL-19 demonstrated robust in vitro expansion and effectively facilitated the infiltration of dendritic and T cells into tumor tissues [209]. These CAR-T cells exhibited significant anti-tumor activity in models of hepatocellular carcinoma, pancreatic cancer, and ovarian cancer [210]. However, further validation is required regarding their efficacy in CNS tumors. Overall, these findings suggest that cytokine-secreting CAR-T cells may hold considerable potential and offer novel strategies to enhance therapeutic efficacy in CNS tumors. However, current research has several limitations. Firstly, the number of relevant experimental studies is relatively limited, with the majority still in the preclinical research phase, leading to clinical trial outcomes that remain unvalidated. Furthermore, the operational procedures for patients have not been standardized, which may negatively impact the safety and efficacy of clinical applications. Therefore, to advance the application of this therapy in clinical practice, there is an urgent need for more in-depth and systematic research to elucidate its mechanisms of action, optimize dosing regimens, and comprehensively assess its clinical efficacy.

### Use of stem-like T cells

Upon antigen stimulation, naïve T cells (TN) rapidly proliferate and differentiate into various subpopulations, including stem-like memory T cells (TSCM), TCM, TEM, and effector T cells (Teff), working synergistically to eliminate infected or cancerous cells. Among these, TSCM and TCM are particularly significant in CAR-T cell engineering owing to their extended lifespan and self-renewal capacities [211]. CAR-T cells generated from TN and TSCM cells (CD62L + CD45RA+) exhibit enhanced expansion potential, reduced exhaustion in animal models, and prolonged tumor suppression. Furthermore, CAR-T cells derived from TN or TSCM cells markedly lower the incidence of CRS and ICANS compared to conventional CAR-T cells [212], suggesting improved efficacy and a better safety profile. These findings highlight the essential role of CAR-T cells with central memory or stem-like memory phenotypes in ensuring long-term survival and sustained anti-tumor activity, offering promising directions for optimizing CAR-T cell therapy. At the same time, it is essential to address the challenge of generating large quantities of memory T cells, as their scarcity poses a significant hurdle for clinical applications. Sabatino M et al. developed a clinical-grade method for generating tumor-redirected TSCM cells. In the presence of IL-7, IL-21, and the glycogen synthase 3β inhibitor TWS119, CD8 + CD62L + CD45RA + TN cells were enriched and activated via CD3/CD28 co-stimulation. The study demonstrated that TSCM-modified CD19 CAR-T cells elicited enhanced anti-tumor responses relative to conventional CD19 CAR-T cells [213]. This clinical-grade selection method provides a basis for assessing the outcomes of clinical trials. However, comprehensive in vivo data and detailed toxicity analyses are still required. Moreover, due to significant variability in TN/SCM cell counts among patients, CAR-T cell manufacturing might necessitate customization based on different tumor types [214]. Therefore, considering both the potential advantages and limitations, whether this approach can effectively translate into clinical advancements requires further validation and investigation.

### Optimising targets

Heterogeneity and antigen escape in CNS tumors are critical factors impacting CAR-T cell efficacy, Therefore, target optimization is crucial for enhancing therapeutic outcomes. Addressing the heterogeneity of antigen expression in CNS tumors necessitates the identification and selection of novel, effective targets. Moreover, employing multi-target CAR-T cells can effectively counteract antigen escape. For example, dual-target CAR-T cells (e.g., targeting EGFR and IL-13Rα2 [150], targeting EGFRvIII and wide-type EGFR protein [215] ) and triple-target CAR-T cells (e.g., targeting EphA2, IL-13Rα2, and EGFRvIII [154]) have demonstrated promising potential in glioma therapy. For patients with CNSL, CD19/CD22 dual-target CAR-T cells and CD19/CD20 dual-target CAR-T cells exhibit significant potential [184]. However, although CNSL and gliomas belong to the category of CNS tumors, there are notable differences in their pathological mechanisms and antigen targets. Therefore, the application of different multi-target strategies in these two types of tumors needs to be distinguished. In summary, precision therapeutic strategies for CNS tumors require in-depth research to fully evaluate the advantages and limitations of multi-target CAR-T cells. By optimizing target selection and designing flexible multi-target CAR-T cells, there is potential to significantly enhance therapeutic effects against various types of CNS tumors. This will provide more effective options for the clinical treatment of CNS tumors and promote advancements in clinical applications in this field.

### Improving CAR-T cell delivery pathways

Intravenous injection is the predominant delivery route in CAR-T cell therapy. However, intravenous injection faces efficacy limitations in CNS tumors owing to the presence of the BBB. In contrast, intracerebroventricular and intra-tumoral injections offer distinct advantages. These local delivery modalities require fewer CAR-T cells and mitigate the significant elevation of inflammatory cytokines, such as IL-10 and IFN-γ, thereby further reducing associated side effects. In addition, these approaches significantly enhance the tumor infiltration capacity of T cells. Locally delivered IL-13Rα2 [102] and HER2 CAR-T cells [110] demonstrated favorable tolerability and positive clinical outcomes in glioma treatment. Although local delivery methods, including intracerebroventricular and intra-tumoral injections, present distinct advantages in CAR-T cell therapy for CNS tumors, their generalizability requires further validation, particularly concerning their applicability across diverse patient populations and tumor types. Furthermore, local delivery involves invasive procedures that may introduce potential side effects, including intracranial infections or inflammation at the injection site. Current data on long-term efficacy are insufficient, necessitating additional clinical trials to validate treatment outcomes. Therefore, further research is essential for a comprehensive assessment of the potential benefits and risks associated with local delivery methods.

**Fig. 4:**
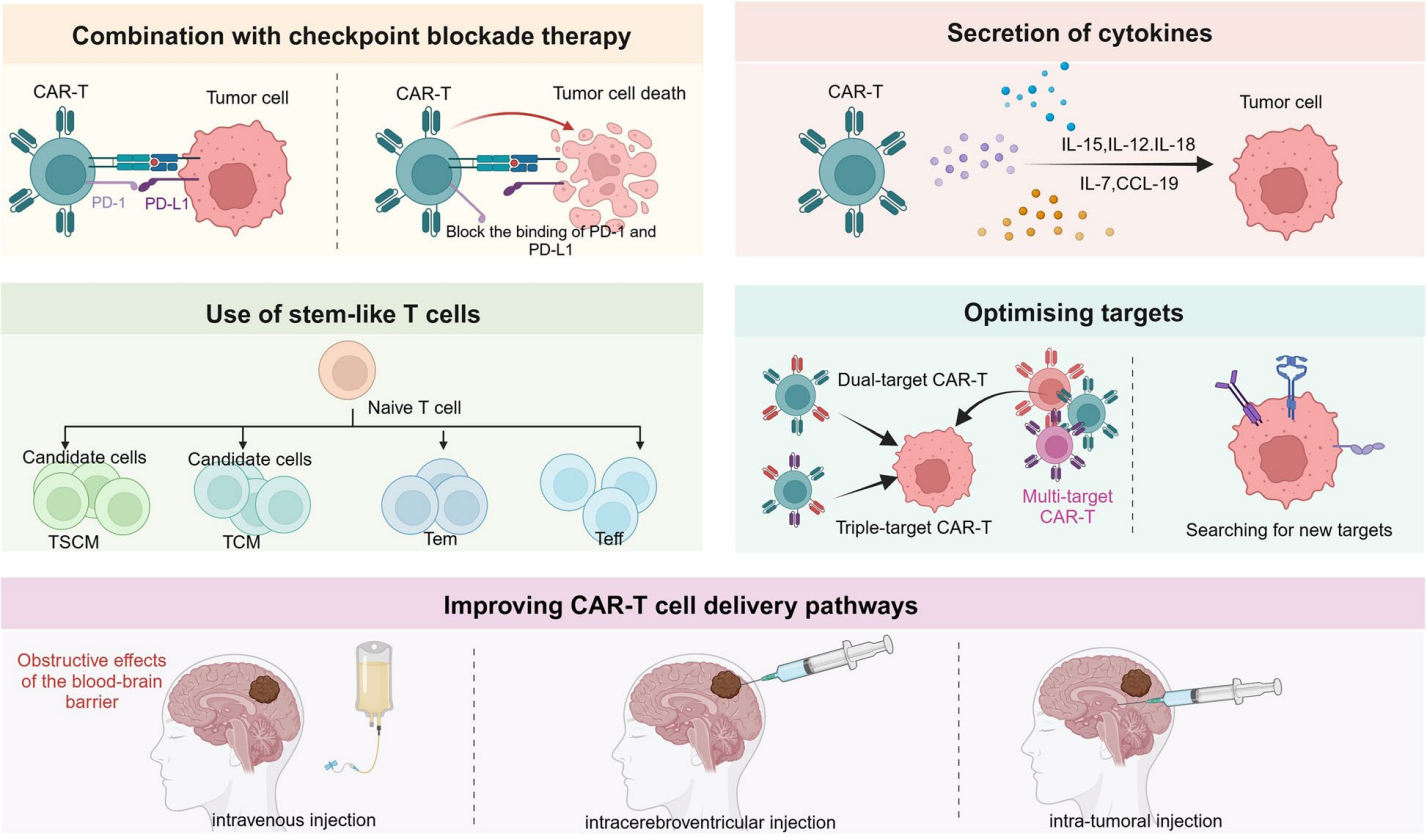
Strategies to enhance CAR-T cell therapy for CNS tumors. Combination with checkpoint blockade therapy: blocking the interaction between PD-1 on CAR-T cells and PD-L1 on tumor cells, preventing T cell exhaustion and enhancing antitumor activity. Secretion of cytokines: CAR-T cells secrete cytokines such as IL-12, IL-15, IL-18, IL-7, and CCL-19 to improve T cell proliferation and tumor infiltration. Use of stem-like T cells: Incorporating stem-like (TSCM) and central memory (TCM) T cells in CAR-T therapy, which have self-renewal and long-term antitumor potential. Optimising targets: Targeting tumor cells with multiple antigens to overcome antigen heterogeneity and prevent tumor escape. Improving CAR-T cell delivery pathways: CAR-T cells are delivered through intravenous routes (with challenges posed by the blood-brain barrier) and local administration methods such as intraventricular or intra-tumoral injections, increasing T cell infiltration and reducing systemic side effects

These strategies have significantly enhanced the overall efficacy of CAR-T cell therapy for CNS tumors at various levels, offering valuable guidance for future clinical applications and research (Fig. 4).

## Conclusion

CAR-T cell therapy has demonstrated substantial potential in treating CNS tumors but continues to encounter numerous challenges. Key factors influencing CAR-T therapeutic efficacy include the BBB, the immunosuppressive nature of the TME, antigen expression heterogeneity and toxicities. Significant progress has been achieved in research on gliomas, MB, and lymphohematopoietic tumors involving the CNS, particularly regarding the targeting of antigens such as EGFRvIII, HER2, B7H3, GD2, and CD19. However, additional research is necessary to optimize therapy design, enhance the ability to traverse the BBB, and address challenges related to immunosuppression and antigenic heterogeneity to facilitate a broader application of CAR-T therapies for CNS tumors. Future research should emphasize the development of multi-targeted CAR-T therapies, localized therapeutic approaches, and combination therapies with other agents, aiming to improve efficacy, minimize side effects, and offer more effective solutions for CNS tumors.

## Data Availability

No datasets were generated or analysed during the current study.

## Abbreviations

CAR-T: Chimeric antigen receptor T-cell
CNS: Central nervous system
TME: Tumor microenvironment
BBB: Blood-brain barrier
MRP: Multidrug resistance protein
BCRP: Breast cancer resistance protein
TAMs: Tumur-associated macrophages
Tregs: Regulatory T cells
MDSCs: Myeloid-derived suppressor cells
ECM: Extracellular matrix
GBM: Glioblastoma
CTL: Cytotoxic T lymphocyte
DC: Dendritic cell
NK: Natural killer
NO: Nitric oxide
ROS: Reactive oxygen species
PNT: Peroxynitrite
CXCR: C-X-C chemokine receptor type
CXCL: C-X-C motif chemokine ligand
CCR: C-C chemokine receptor type
CCL: C-C motif chemokine ligand
CTLA-4: Cytotoxic T-lymphocyte-associated protein 4
TGF-β: Transforming growth factor-beta
IL-10: Interleukin-10
TSAs: Tumor-specific antigens
TAAs: Tumor-associated antigens
EGFRvIII: Epidermal growth factor receptor variant III
CRS: Cytokine release syndrome
ICANS: Immune effector cell-associated neurotoxicity syndrome
GM-CSF: Granulocyte-macrophage colony-stimulating factor
CSF: Cerebrospinal fluid
IFN-γ: Interferon-γ
DMG: Diffuse midline glioma
DIPG: Diffuse intrinsic pontine glioma
VEGF: Vascular endothelial growth factor
rGBM: Recurrent glioblastoma
IDO1: Indoleamine 2,3-dioxygenase 1
PFS: Progression-free survival
OS: Overall survival
scFv: Single-chain variable fragment
HGG: High-grade glioma
MRI: Magnetic resonance imaging
SD: Stable disease
PR: Partial remission
CR: Complete remission
ZFN: Zinc finger nucleases
HER2: Human epidermal growth factor receptor 2
VST: Virus-specific T cells
4S CAR: Fourth-generation CAR-T cells
PD: Progressive disease
IL-7R: IL-7 receptor
TIAN: Tumor inflammation-associated neurotoxicity
VEGFA: Vascular endothelial growth factor A
TMIGD2: Transmembrane and immunoglobulin domain–containing 2
CSPG4: Chondroitin sulfate proteoglycan 4
GBM-NS: Glioblastoma neurosphere
NKG2D: Natural killer cell group 2 member D
NKG2DL: Natural killer cell group 2 member D Ligand
MHC: Major histocompatibility complex
ULBPs: UL16-binding proteins
TCM: Central memory T cells
TEM: Effector memory T cells
CSCs: Tumor stem cells
EPCs: Endothelial progenitor cells
MB: Medulloblastoma
PFA: Posterior fossa A
TM: Transmembrane
iC9: Inducible caspase 9
PDOX: Patient derived orthotopic xenograft
CNSL: Central nervous system lymphoma
PCNSL: Primary central nervous system lymphoma
SCNSL: Secondary central nervous system lymphoma
WBRT: Whole-brain radiotherapy
ORR: Overall response rate
RFS: Relapse-free survival
DLBCL: Diffuse large B-cell lymphoma
R/R CNSL: Relapse/refractory CNSL
B-ALL: Acute B-cell lymphoblastic leukemia
EFS: Event-free survival
LFS: Leukemia-free survival
NHL: Non-hodgkin lymphoma
AML: Acute myeloid leukemia
MM: Multiple myeloma
AL: Acute leukemia
mAbs: Monoclonal antibodies
ADCs: Antibody-drug conjugates
DoR: Duration of response
PD-L1: Programmed death ligand 1
PD-1: Programmed death 1
B2M: β2 microglobulin
TN: Naïve T cells
TSCM: Stem-like memory T cells
Teff: Effector T cells
